# First-line treatments for extensive-stage small-cell lung cancer with immune checkpoint inhibitors plus chemotherapy: a China-based cost-effectiveness analysis

**DOI:** 10.3389/fimmu.2024.1408928

**Published:** 2024-07-05

**Authors:** Lidan Yi, Zhen Zhou, Xiaohui Zeng, Chongqing Tan, Qiao Liu

**Affiliations:** ^1^ Department of Pharmacy, The Second Xiangya Hospital of Central South University, Changsha, China; ^2^ School of Public Health and Preventive Medicine, Monash University, Melbourne, VIC, Australia; ^3^ Positron Emission Tomography - Computed Tomography (PET-CT) Center, The Second Xiangya Hospital of Central South University, Changsha, China

**Keywords:** cost-effectiveness, ES-SCLC, PD-L1, ICIs, adebrelimab, serplulimab

## Abstract

**Objective:**

To determine the cost-effectiveness of imported immune checkpoint inhibitors (ICIs) such as atezolizumab and durvalumab, and domestic ICIs like serplulimab and adebrelimab, in combination with chemotherapy for extensive-stage small cell lung cancer (ES-SCLC) in China.

**Methods:**

Using a 21-day cycle length and a 20-year time horizon, a Markov model was established to compare the clinical and economic outcomes of five first-line ICIs plus chemotherapy versus chemotherapy alone, as well as against each other, from the perspective of the Chinese healthcare system. Transition probabilities were estimated by combining the results of the CAPSTONE-1 trial and a published network meta-analysis. Cost and health state utilities were collected from multiple sources. Both cost and effectiveness outcomes were discounted at a rate of 5% annually. The primary model output was incremental cost-effectiveness ratios (ICERs). A series of sensitivity analyses were preformed to assess the robustness of the model.

**Results:**

In the base-case analysis, the addition of first-line ICIs to chemotherapy resulted in the ICERs ranged from $80,425.31/QALY to $812,415.46/QALY, which exceeded the willing-to-pay threshold set for the model. When comparing these first-line immunochemotherapy strategies, serplulimab plus chemotherapy had the highest QALYs of 1.51286 and the second lowest costs of $60,519.52, making it is the most cost-effective strategy. Our subgroup-level analysis yielded results that are consistent with the base-case analysis. The sensitivity analysis results confirmed the validity and reliability of the model.

**Conclusion:**

In China, the combination of fist-line ICIs plus chemotherapy were not considered cost-effective when compared to chemotherapy alone. However, when these fist-line immunochemotherapy strategies were compared with each other, first-line serplulimab plus chemotherapy consistently demonstrated superiority in terms of cost-effectiveness. Reducing the cost of serplulimab per 4.5 mg/kg would be a realistic step towards making first-line serplulimab plus chemotherapy more accessible and cost-effective.

## 1 Introduction

China carried a high burden of lung cancer, contributing to about two-fifths of the global lung cancers (1, 2). Small cell lung cancer (SCLC) is a subtype of lung cancer that accounts for around 15 percent of all diagnosed cases. SCLC is known to be highly aggressive due to its rapid proliferation and metastasis (3). Approximately two-thirds of SCLC cases are diagnosed at an extensive-stage [ES] disease (4). In the preimmunotherapy era, the standard first-line treatment for ES-SCLC was chemotherapy with a platinum-based agent plus etoposide (5). Unfortunately, the prognosis for patients with ES-SCLC treated with chemotherapy alone is generally poor, with a median overall survival (OS) of around 10 months and a 2-year survival rate typically below 5% (6, 7). In recent years, immune checkpoint inhibitors (ICIs) have demonstrated promising results in improving outcomes for patients with ES-SCLC. When ICIs are added to traditional chemotherapy, they have been shown to extend the median overall survival (OS) of ES-SCLC patients to approximately 12 to 15 months (8–12). As a result, the use of immunochemotherapy has gradually emerged as the mainstay of first-line treatment for ES-SCLC.

Up to now, the Chinese National Medical Products Administration (NMPA) have successively approved 4 ICIs combined chemotherapy for the first-line treatment of ES-SCLC, including two ICIs (atezolizumab and durvalumab) (13, 14), and two domestic ICIs (serplulimab and adebrelimab) (15, 16). While the introduction of innovative ICIs for the treatment of ES-SCLC in China has brought notable clinical benefits, it has also imposed a significant economic burden on both individual patients and the Chinese government. The annual costs of using these ICIs, as estimated based on the latest bid-winning prices (17), ranges from $40,000 to 140,000. These costs are considerably higher than the China’s per capita gross domestic product (GDP) of $12,681 in 2023 (18). Given the lager population of beneficiaries and the potential negative financial consequences, comparing the cost-effectiveness of the approved immunochemotherapy options among Chinese patients with ES-SCLC is crucial to determine their appropriateness for widespread clinical use. While one existing China-based study have evaluated the cost-effectiveness of ICIs for ES-SCLC (19), it is important to acknowledge potential limitations may impact the generalizability of their findings to real-world settings: firstly, this study included ICIs (such as pembrolizumab, ipilimumab, and nivolumab) that have not yet been officially authorized by NMPA for first-line treatment of ES-SCLC, which may limit the relevance and the value of the study finding for patients and clinicians; secondly, this study was unable to report on the cost-effectiveness serplulimab and adebrelimab due to their recent approval dates (January 16, 2023 and February 28, 2023, respectively), which may not accurately reflect the current advancement of clinical treatment.

To provide the up-to-data pharmacoeconomics evidence for clinical decision-making, we conducted this study to compare the cost-effectiveness of all approved ICIs combined chemotherapy as the first-line treatment for ES-SCLC patients from the perspective of the Chinese healthcare system.

## 2 Material and methods

### 2.1 Overview

This study established a Markov model to comparing the cost-effectiveness of six competing first-line treatment strategies for Chinese patients with ES-SCLC. The strategies evaluated in the study were as follows:

1. Atezolizumab plus chemotherapy.
2. Durvalumab plus chemotherapy.
3. Durvalumab plus tremelimumab plus chemotherapy.
4. Serplulimab plus chemotherapy.
5. Adebrelimab plus chemotherapy.
6. Chemotherapy alone.

We incorporated a chemotherapy arm in the model, as chemotherapy remains one of the preferred first-line treatment options recommended by the Chinese Society of Clinical Oncology for ES-SCLC (5). In this study, chemotherapy was modeled as etoposide plus carboplatin, as this combination is commonly used in current clinical practice in China for the first-line treatment of ES-SCLC. Information on the relative clinical efficacy and safety data for these six competing first-line treatment strategies was collected from a recently published network meta-analysis (NMA) conducted by Wang S et al. (20), as there were no clinical trial directly comparing the clinical performance of these strategies as first-line options for ES-SCLC patients. Meanwhile, we obtained costs and health state utilities from multiple sources, including national publicly available databases, local hospitals and previous literature. Target patients was patients aged 18 years or older with treatment-naive, histologically or cytologically documented ES-SCLC.

This study was designed in accordance with the China Guidelines for Pharmacoeconomic Evaluations (2020 Edition) (21), and reported according to the Consolidated Health Economic Evaluation Reporting Standards (CHEERS) reporting guideline (22). Since this study solely utilized existing, non-identifiable data and did not involve any direct interaction or intervention with human subjects, it was deemed exempt from obtaining approval of the Chinese Ethics Review Committee.

### 2.2 Model construction

**Figure 1:**
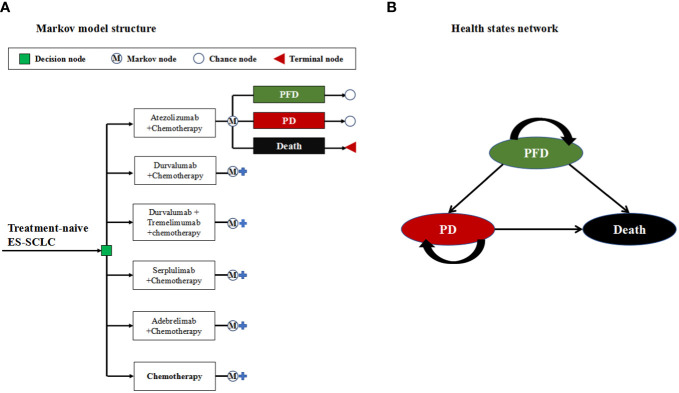
**(A)** Markov model structure used to six competing first-line treatment strategies for Chinese patients with ES-SCLC. **(B)** Health states network showing the possible transitions between 3 mutually exclusive health states. ES-SCLC, extensive-stage small cell lung cancer; PFD, progression-free disease; PD, progressed disease.

To simulate the clinical progression trajectory of ES-SCLC in the Markov model, three mutually exclusive health states were constructed: progression-free disease (PFD) health state, progressed disease (PD) health state and death (**Figure 1**). The Markov cycle length was set to align with the treatment administration interval specified in the phase III clinical studies referenced (8–12), which was 21 days. This model assumed that all patients start in initial health state of PFD, and were randomly assigned one of six first-line treatment strategies being evaluated (Details information on dosage and administration schedule for first-line were provided in **Supplementary Table S1**). During each Markov cycle, patients would stay in the PFD health state if they did not experience disease progression or death. However, if disease progression occurred during a particular cycle, patients would transition to the PD health state. Alternatively, if death from ES-SCLC occurred during a cycle, patients would enter the terminal health state. Patients in the PD health state would be eligible to receive subsequent anticancer therapies. In addition to receiving anticancer therapies, patients would also be supplemented with best supportive care (BSC) (5). Moreover, the model considers the provision of palliative care before imminent death (5). The Markov model was designed with a 20-year time horizon in the study, guided by the longest median OS of 15.4 months observed in ES-SCLC patients receiving first-line ICIs combined with chemotherapy (11). This duration was chosen to ensure that nearly all model patients would progress to the terminal state (death health state).

This cost-effectiveness analysis was conducted from the perspective of the Chinese healthcare system, which refers to weighing the consumption of healthcare resources against the clinical benefits of interventions obtained by patients in the context of the national healthcare system (23). We reported medical costs for each strategy in 2023 US dollars (1 United States dollars is equivalent to 7.0467 Chinese yuan), and measured their effectiveness in quality adjusted life years (QALYs). Both costs and effectiveness were discounted at an annual rate of 5%. The primary model output were incremental cost-effectiveness ratios (ICERs), which represents the ratio of the incremental medical costs to the incremental QALYs gained between two treatment strategies.

### 2.3 Transition probability

Theoretically, the transition probabilities of first-line chemotherapy were estimated from the Kaplan-Meier (KM) survival curves published in the CAPSTONE-1 study, because it is the only phase III clinical trial investigating the efficacy and safety profiles of the ICIs plus chemotherapy as a first-line treatment for Chinese patients with ES-SCLC; while the transition probabilities of five first-line ICI-based treatment were estimated based on pooled hazard ratios (HRs) provided by the aforementioned NMA.

Initially, for the first-line chemotherapy, we digitized the PFS and OS data from KM curves to create pseudo-individual patient data (24). Subsequently, a series of goodness-of-fit tests were conducted to identify the optimal parametric survival distribution for these reconstructed data. These tests involved evaluating statistical metrics such as the Akaike information criterion (AIC) and Bayesian information criterion (BIC) and comparing modeled curves with KM curves graphically. Lower AIC and BIC values indicate a better fit, with increased overlap between the modeled and KM curves signifying a stronger fit. The results of the goodness-of-fit tests, including AIC and BIC values, were detailed in **Supplementary Table S2**, with graphical assessments provided in **Supplementary Figures S1-S2**. Ultimately, the log-logistic distribution was selected to fit and extrapolate the survival outcomes of first-line chemotherapy. The parameters theta (θ) and kappa (κ) were utilized to calculate transition probabilities between health states. The survival probabilities at a specific time cycle (t) were determined as follows: $S(t)=1/[1+exp(\theta )t^{\kappa }]$ .

For these five first-line ICIs plus chemotherapy, we first obtained the HRs of their PFS and OS compared to chemotherapy alone. Then their survival probabilities were adjusted using a specific formula: $S{\left(t\right)}_{ICIs+chemo}=1/{\left[1+exp(\theta )t^{\kappa }\right]}^{HR}$ . Analogically, the HRs estimated for different subgroups based on gender, age and baseline Eastern Cooperative Oncology Group (ECOG) performance status scores were used to estimated subgroup-level transition probabilities. **Supplementary Table S3** listed model inputs related to the estimation of transition probabilities.

### 2.4 Costs and utilities

This model considered various medical expenditures associated with fist-line drug acquisition, AEs management, subsequent anticancer therapies, routine follow-up, BSC and palliative care.

The drug acquisition costs of first-line drugs were accumulated by cycles. First, we retrieved the latest bid-winning prices for tezolizumab, durvalumab, adebrelimab, serplulimab, etoposide from the National Health Industry Data Platform (17), and the current market prices in Hong Kong for tremelimumab (25). Then, the costs of these drugs per cycle were calculated based on the administration dosages per cycle listed in **Supplementary Table S1**. For drugs with anthropometry-dependent dosages, the targeted patients were modeled as having an average weight of 69.6 kg for male and 59.0 kg for female (26), an average body surface area of 1.72 m^2^ and an average creatinine clearance rate of 70 ml/min (27). Given the absence of direct safety comparison data for the six first-line strategies under evaluation, the medical costs associated with managing AEs were estimated through the following steps Step 1: Determination of charging items: Charging items for treating each AE associated with anticancer drug treatment were identified based on either Chinese expert consensus or oncologists’ opinions (28–35), with relevant costs collected from local general hospitals *(* **Supplementary Table S4** *)*; Step 2: Estimation of AEs management cost for first-line chemotherapy: The AEs management cost for first-line chemotherapy was calculated by multiplying the frequency of grades 3/4 AEs by the estimated cost for each AE. Step 3, Estimation of AEs management cost for first-line ICIs plus chemotherapy: HRs for grades 3/4 AEs derived from the NMA mentioned in the study were used to estimate the AEs management cost for the five first-line ICIs plus chemotherapy strategies. Additionally, medical costs related to subsequent anticancer therapies, routine follow-up, BSC and palliative care were obtained from published literature (27).

Chinese-specific health state utilities were utilized in the model, with assigned scores of 0.856 and 0.768 for the PFD and PD health states, respectively (36). The negative effects of grades 3/4 AEs resulting from first-line treatments on health state utilities were computed as frequency-weighted sums using the same methodology employed for estimating AE management costs. To conduct this calculation, we sourced the disutility for each AE from the Institute for Clinical and Economic Review (37) and the duration for each AE from published papers (38–42) *(* **Supplementary Table S5** *).* The calculation of grades 3/4 AEs-induced costs and utilities for each first-line treatment was detailed in **Supplementary Table S6**. All model inputs related to costs and health state utilities estimation were summarized in **Supplementary Table S7**.

### 2.5 Statistical analysis

#### 2.5.1 Base-case ICERs

The statistical tools used in this cost-effectiveness analysis included treeAge Pro Healthcare software (version 2022, https://www.treeage.com/) and R software (version 4.0.4, http://www.r-project.org). The model determined the relative cost-effectiveness between two competing strategies by comparing their ICER with a preset willing-to-pay (WTP) threshold. In the absence of explicit WTP threshold benchmarked established for ICER-based decisions in China, the study followed the recommendations provided by Cai et al. (18). We utilized a range of 1.2 to 3.0 times China’s GDP in 2022 as the potential WTP threshold, which translated to a value of $15,217.00 to $38,042.49/QALY (18). A strategy with an ICER below the predetermined WTP threshold is considered cost-effective. otherwise is considered non cost-effective.

#### 2.5.2 Subgroup-level ICERs

The subgroup-level HRs of OS for five first-line ICIs plus chemotherapy versus chemotherapy alone by gender, age and baseline ECOG performance status scores, were used to explore the cost-effectiveness results for first-line ICIs plus chemotherapy at a subgroup-level.

#### 2.5.3 Sensitivity analysis

Two sensitivity analyses were performed to assess and validate the robustness of the cost-effectiveness results obtained. In the deterministic sensitivity analyses (DSA) performed, we identified the impact of the uncertainty associated with individual model input on the model by varying its value within plausible ranges, such as 95% confidence intervals for HRs, 0%~8% for discount rate and baseline values plus or minus 25% for other model inputs. When conducting probabilistic sensitivity analyses (PSA), we investigate the influence of the uncertainties in multiple model inputs on the findings with 10,000 Monte Carlo simulations. During each Monte Carlo simulation, model inputs were randomly sampled from the appropriate distributions recommended by the ISPOR-SMDM Modeling Good Research Practices Task Force (43). The ranges for DSA and distributions for PSA were outlined in **Supplementary Table S3** and **Supplementary Table S7**.

## 3 Results

### 3.1 Base-case ICERs

In the cohort of Chinese patients with ES-SCLC, the addition of first-line ICIs to chemotherapy resulted in cost increments ranging from $38,825.40 to $134,536.88 and survival enhancements from 0.16560 to 0.48629 QALYs (equivalent to 2.0 to 5.8 months), as detailed in **Table 1**. Consequently, the calculated ICERs ranged from $80,425.31/QALY to $812,415.46/QALY, surpassing the model’s WTP threshold. This indicated that none of the strategies involving ICIs in combination with chemotherapy were deemed cost-effective compared to chemotherapy alone.

**Table 1:** Base-case and stepwise ICERs comparison of first-line treatment strategies.

ICERs (vs chemotherapy)					
Strategy	Costs (US$)	QALYs	Incremental costs	Incremental QALYs	ICER ($/QALY)
Chemotherapy	21,409.67	1.02657	NA	NA	NA
Adebrelimab+Chemotherapy	60,235.07	1.33257	38,825.40	0.30600	126,879.72
Serplulimab+Chemotherapy	60,519.52	1.51286	39,109.85	0.48629	80,425.31
Atezolizumab+Chemotherapy	73,236.05	1.26971	51,826.38	0.24314	213,157.14
Durvalumab+Chemotherapy	90,534.88	1.28249	69,125.20	0.25592	270,108.50
Durvalumab+Tremelimumab+Chemotherapy	155,946.55	1.19217	134,536.88	0.16560	812,415.46
Stepwise ICERs comparison					
Strategy^a^	Costs (US$)	QALYs	Incremental costs^b^	Incremental QALYs^b^	ICER ($/QALY)^c^
Chemotherapy	21,409.67	1.02657	NA	NA	NA
Adebrelimab+Chemotherapy	60,235.07	1.33257	38,825.40	0.30600	126,879.72 **(ED)**
Serplulimab+Chemotherapy	60,519.52	1.51286	284.45	0.18029	1,577.76
Atezolizumab+Chemotherapy	73,236.05	1.26971	12,716.54	-0.24315 **(D)**	-52,298.96
Durvalumab+Chemotherapy	90,534.88	1.28249	17,298.82	0.01278	1,353,642.30
Durvalumab+Tremelimumab+Chemotherapy	155,946.55	1.19217	65,411.67	-0.09032 **(D)**	-724,258.63
Excluding dominated and extended dominated strategies:					
Chemotherapy	21,409.67	1.02657	NA	NA	NA
Serplulimab+chemotherapy	60,519.52	1.51286	39,109.85	0.48629	80,425.31
Durvalumab+Chemotherapy	90,534.88	1.28249	30,015.36	-0.23037 **(D)**	-130,291.19
Excluding extended dominated strategies:					
Chemotherapy	21,409.67	1.02657	NA	NA	NA
Serplulimab+chemotherapy	60,519.52	1.51286	39,109.85	0.48629	80,425.31

QALYs, quality-adjusted life-years; ICERs, incremental cost-effectiveness ratios; NA, not applicable; D, dominated strategy(a strategy is less effective and more costly than its previous alternative strategy). ED, extended dominated strategy (a strategy is less effective and less cost-effective than its next alternative strategy).

a For a stepwise ICER comparison, all competitive strategies are arranged in ascending order of cost.

b The increment costs and QALYs were calculated as the differences between the current strategy and its previous alternative.

c During each round of comparison, once dominated and extended dominated strategies are identified, they are excluded from the next round of comparison.

Upon conducting a stepwise ICER comparison of five first-line immunochemotherapy regimens, it was revealed that first-line serplulimab combined with chemotherapy exhibited the highest QALYs at 1.51286 and the second-lowest costs at $60,519.52, positioning it as the most cost-effective strategy (refer to **Table 1**). Conversely, the analysis indicated that the first-line utilization of durvalumab in conjunction with tremelimumab and chemotherapy resulted in the lowest QALYs at 1.19217 and the highest costs at $155,946.55, highlighting it as the least cost-effective approach.

### 3.2 Subgroup-level ICERs

The results presented in **Supplementary Table S8** reveals that the comparison between first-line ICIs combined with chemotherapy and chemotherapy alone demonstrated significant increases in both costs and survival outcomes across all subgroups:

- Male subgroup: cost increase: $38,536.12 to $134,228.09; survival extension: 0.15439 to 0.46544 QALYs (equivalent to 1.9 to 5.6 months).
- Female subgroup: cost increase: $43,041.79 to $136,596.00; survival extension: 0.24036 to 0.62921 QALYs (corresponding to 2.9 to 7.6 months).
- Age≥65 subgroup: cost increase: $39,708.64 to $131,804.96; Survival extension: 0.06645 to 0.55656 QALYs (equivalent to 0.8 to 6.7 months).
- Age<65 subgroup: cost increase: $39,259.64 to $136,596.00; Survival extension: 0.05239 to 0.50792 QALYs (corresponding to 0.6 to 6.1 months).
- ECOG PS of 0 subgroup: cost increase: $34,857.36 to $135,519.68; survival extension: 0.16182 to 1.08791 QALYs (equivalent to 1.9 to 13.1 months).
- ECOG PS of 1 subgroup: cost increase: $37,982.82 to $133,077.26; survival extension: 0.11262 to 0.44533 QALYs (corresponding to 1.4 to 5.4 months).

Overall, the use of first-line ICIs plus chemotherapy resulted in higher costs compared to chemotherapy alone, leading to inferior cost-effectiveness despite the survival benefits achieved.

Among the five first-line ICIs plus chemotherapy regimens compared, serplulimab plus chemotherapy provided the best survival outcomes for most subgroups, except for the Age ≥ 65 subgroup where atezolizumab plus chemotherapy achieved the highest survival (1.58313 QALYs). The QALYs achieved with serplulimab plus chemotherapy were as follows:

- Male subgroup: 1.49201 QALYs.
- Female subgroup: 1.65578 QALYs.
- Age ≥ 65 subgroup: 1.58025 QALYs.
- Age< 65 subgroup: 1.53449 QALYs.
- ECOG performance status score of 0 subgroup: 2.11448 QALYs.
- ECOG performance status score of 1 subgroup: 1.47190 QALYs.

Additionally, serplulimab plus chemotherapy proved to be the most cost-effective option in all subgroups due to its relatively lower costs.

### 3.3 Sensitivity analysis

**Figure 2:**
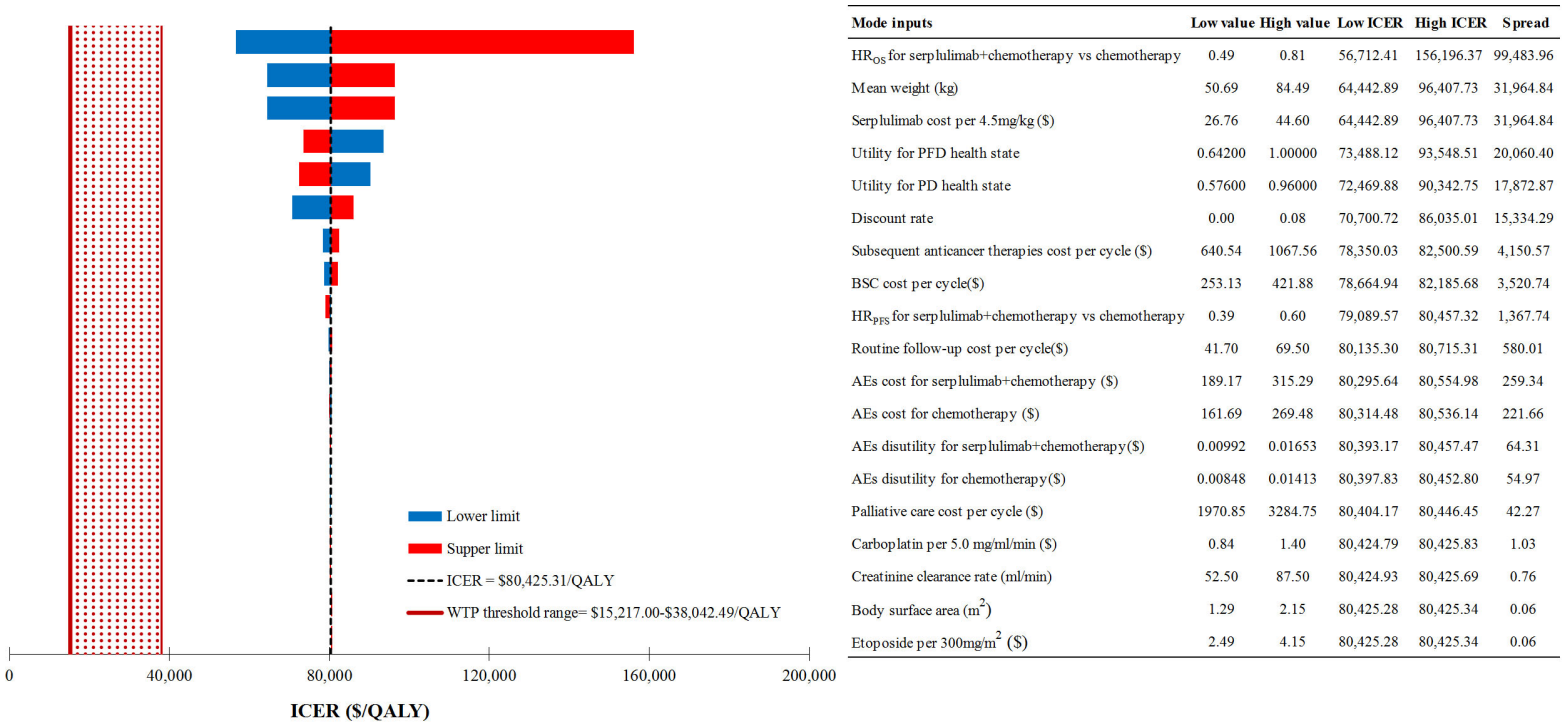
Deterministic sensitivity analysis results for first-line serplulimab+chemotherapy vs chemotherapy alone. ICER, incremental cost-effectiveness ratio; WTP, willingness-to-pay; QALY, quality-adjusted life-year; HR, hazard ratio; OS, overall survival; PFS, progression-free survival; PFD, progression-free disease; PD, progressed disease; BSC, best supportive care; AEs, adverse events.

The study identified first-line serplulimab in combination with chemotherapy as the most cost-effective option among five different combinations of ICIs and chemotherapy. Consequently, our DSA focused on comparing the efficacy of first-line serplulimab plus chemotherapy versus chemotherapy alone. The DSA results (**Figure 2**) highlighted that the HR of OS comparing serplulimab plus chemotherapy to chemotherapy had the most substantial impact on the ICER, with patients’ mean weight and the cost of serplulimab per 4.5mg/kg following in influence. Detailed results of further one-way sensitivity analyses for these key factors were presented in **Supplementary Figures S3** ***-*** **S5**. Despite variations in these parameters, the cost-effectiveness of serplulimab plus chemotherapy remained unchanged.

**Figure 3:**
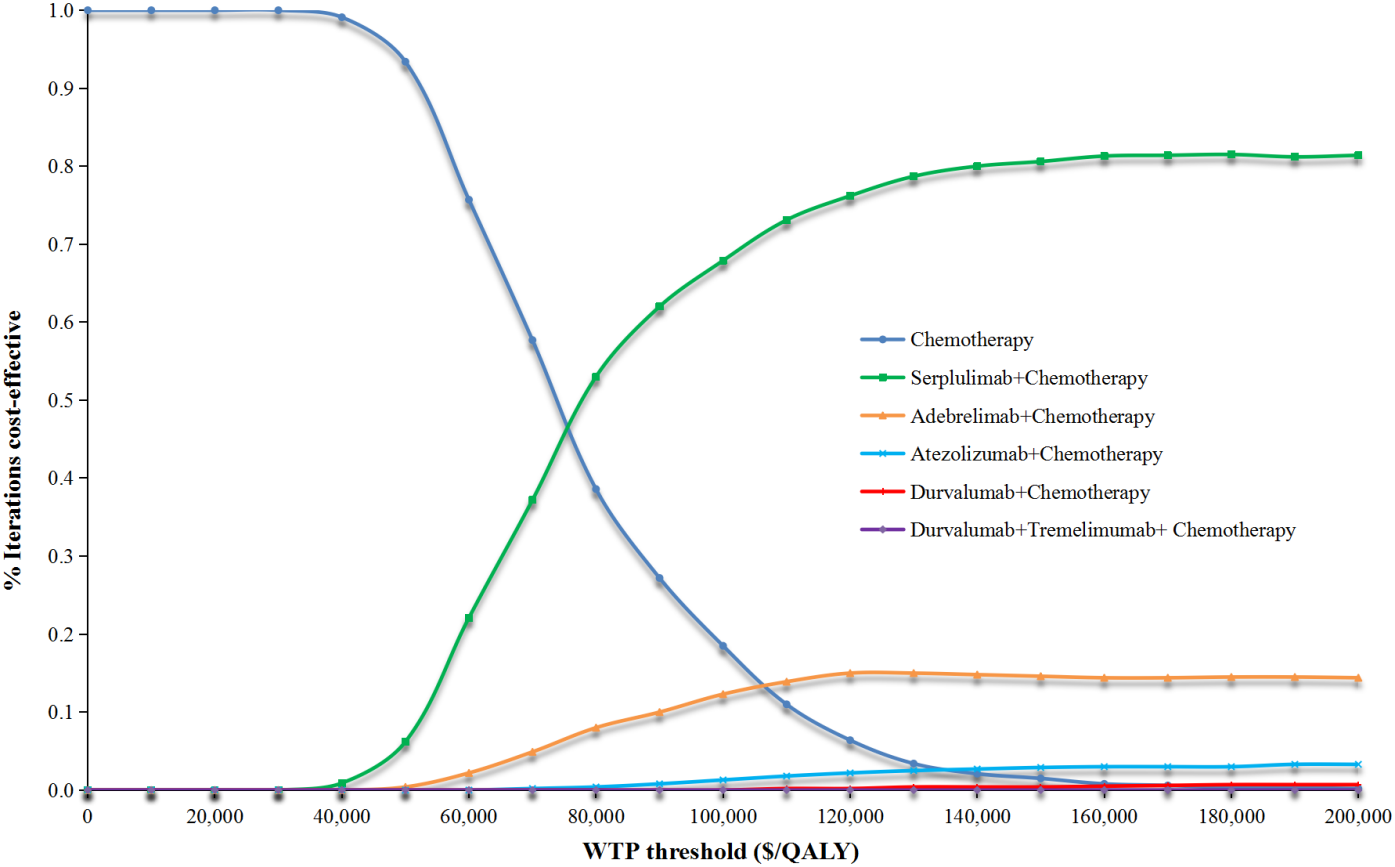
Probabilistic sensitivity analyses results for overall Chinese patients with ES-SCLC. WTP, willingness-to-pay; ES-SCLC, extensive-stage small cell lung cancer.

The PSA results were displayed through cost-effectiveness acceptability curves, including **Figure 3** for the overall patient population and **Supplementary Figures S6** ***-*** **S11** for specific subgroups. Notably, as the WTP threshold increased, the likelihood of first-line serplulimab plus chemotherapy being cost-effective was most prominent compared to the other first-line ICI plus chemotherapy strategies studied across all subgroups in the analysis.

## 4 Discussion

### 4.1 Principal findings

This research systematically compared the cost-effectiveness of five first-line ICIs-based strategies, which have been approved in recent years in China (13–16), along with the commonly used chemotherapy regimen consisting of etoposide plus carboplatin among Chinese patients with ES-SCLC and aimed to provide valuable insights into the suitable therapy for this patient population. Our study showed that:

- In comparing first-line ICIs plus chemotherapy to chemotherapy alone, our study found that despite the observed survival improvement (ranging from 2 to 6 months) with these combination strategies, the substantial additional costs (ranging from $38,825.40 to $134,536.88) associated with them outweigh their limited benefits. This findings suggested that from a cost-effectiveness standpoint, first-line immunochemotherapies may not represent favorable options compared to chemotherapy alone. This conclusion holds true across all subgroup analyses conducted in our study. These results highlight the importance of striking a balance between the significant potential of immunochemotherapies in improving clinical outcomes in ES-SCLC and the economic burden associated with these treatments.
- In our study comparing the cost-effective among five first-line ICIs plus chemotherapy treatments, we found that serplulimab plus chemotherapy consistently outperforming the alternatives treatment options not only in the overall patient population but also in subgroup analyses. This information implied that serplulimab plus chemotherapy provided a favorable balance between clinical outcomes and associated medical costs, as reflected in its almost highest QALYs and relatively low costs. Furthermore, the PSA results indicated that the probability of first-line serplulimab plus chemotherapy being cost-effective was most pronounced as the WTP threshold increased across all subgroups and the total patient population. This results emphasized the robustness of the cost-effectiveness advantages associated with serplulimab plus chemotherapy and the importance of considering the context-specific WTP when evaluating the economic feasibility of treatment options.
- Results from DSA revealed that the top three model inputs with the greatest impact on the ICER of first-line serplulimab plus chemotherapy relative to chemotherapy alone were HR of OS, the mean weight of patients and the cost of serplulimab per 4.5mg/kg. The HR of OS served as a crucial determinant, as it reflected the relative survival benefit of competing strategies. A lower HR indicates better survival outcomes for patients receiving serplulimab plus chemotherapy. The mean weight of patients, likely determining the dosage of serplulimab required, was another significant factor influencing the ICER. This emphasized the need to consider individualized dosing based on patient characteristics to optimize treatment effectiveness. The cost of serplulimab per 4.5mg/kg also played a notable role in the ICER calculation, as it directly affected the overall medical cost of first-line serplulimab plus chemotherapy. According to the further one-way sensitivity analysis, meeting any of these criteria would achieve the cost-effectiveness for the combination therapy of serplulimab plus chemotherapy: HR of OS< 0.25, patient’s mean weight< 22.78 kg, or the cost of serplulimab per 4.5mg/kg< $12.02. Since the cost of serplulimab per 4.5mg/kg is the only factor that can be influenced through policy, ongoing research can play a crucial role in identifying opportunities for price negotiations or alternative reimbursement models to improve access to serplulimab plus chemotherapy without compromising financial sustainability (44).

### 4.2 Strengths and limitation

This study has several notable strengths that contribute to its significance. Firstly, it stands out for incorporating two recently approved domestic ICIs, serplulimab and adebrelimab, in combination with chemotherapy, a novel approach not explored in previous studies (19, 45). This integration enriches the economic evaluation, offering current pharmacoeconomic evidence that mirrors contemporary clinical practices. Moreover, the observation that domestic ICIs are generally more cost-effective than imported alternatives further underscores the importance of evaluating their efficacy in treating ES-SCLC (46). Secondly, the study was valuable as it not only compared the cost-effectiveness of five first-line ICIs combined with chemotherapy against chemotherapy alone but also evaluated the cost-effectiveness of the five ICIs-based treatments individually among Chinese patients with ES-SCLC. Understanding the relative costs and benefits of each ICI-based treatment compared to both chemotherapy alone and other ICIs could assist decision-makers, policymakers, and healthcare providers to make informed decisions regarding the allocation of resources and selecting optimal treatment strategies. Thirdly, we systematically considered the impact of AEs in the model involved taking into account their negative consequences on both health state utilities and the additional treatment costs incurred. Fourthly, by using local expert-recommended treatment items and locally derived costs, the model can provide valuable insights into the economic impact of AEs within the Chinese healthcare context. Fifthly, the cost-effectiveness analysis also considered six major subgroups to evaluate the results in a more comprehensive manner. Analyzing subgroups allows for a deeper understanding of how these treatments may impact different populations or specific demographic characteristics.

This study also has several limitations. Firstly, the primary challenge lies in the absence of direct comparative data on the efficacy and safety of the six first-line strategies analyzed in the model. To overcome this limitation, the study incorporated findings from an authoritative NMA to enhance the model. However, it is essential to acknowledge that relying on indirect data sources can introduce uncertainties and assumptions into the model. For instance, the model did not consider the duration of exposure to immunotherapy, assumed uniform subsequent anticancer therapies upon entering the PD health state, and calculated the cost and disutility of first-line ICIs plus chemotherapy based on clinical safety data of first-line chemotherapy and HRs for grades 3/4 AEs derived from the NMA. Enhancing our models with more robust data in the future could optimize our approach. Secondly, as specific quality-of-life data for Chinese patients with ES-SCLC was unavailable, the study integrated health state utilities from existing literature assessed in Chinese non-small cell lung cancer patients, potentially introducing uncertainty. However, our DSA revealed that considerable variations in health utilities within an acceptable range did not markedly influence our cost-effectiveness results. This indicates that even with more precise data, our conclusions would remain consistent. Thirdly, the uniqueness of the Chinese health system and economic environment may limit the applicability of our findings to different contexts. Considering that China accounts for about 40% of the world’s lung cancer patients, the results of our study could still hold considerable relevance in reducing the global burden of this disease.

## 5 Conclusion

From the perspective of the Chinese healthcare system, the combination of five first-line ICIs plus chemotherapy were not considered cost-effective when compared to chemotherapy alone. However, when these five first-line ICIs plus chemotherapy were compared with each other, first-line serplulimab plus chemotherapy consistently demonstrated superiority in terms of cost-effectiveness. Reducing the cost of serplulimab per 4.5 mg/kg would be a realistic step towards making first-line serplulimab plus chemotherapy more accessible and cost-effective.

## Data availability statement

The original contributions presented in the study are included in the article/**Supplementary Material**. Further inquiries can be directed to the corresponding author.

## Author contributions

LY: Data curation, Formal Analysis, Funding acquisition, Investigation, Resources, Validation, Writing – original draft. ZZ: Data curation, Formal Analysis, Investigation, Validation, Writing – original draft. XZ: Conceptualization, Methodology, Software, Validation, Visualization, Writing – review & editing. CT: Conceptualization, Methodology, Software, Validation, Visualization, Writing – review & editing. QL: Conceptualization, Methodology, Project administration, Software, Supervision, Validation, Visualization, Writing – review & editing.
