# Supplementary material for: First-line treatments for extensive-stage small-cell lung cancer with immune checkpoint inhibitors plus chemotherapy: a China-based cost-effectiveness analysis
Source: Front Immunol. 2024 Jul 5;15:1408928. doi: 10.3389/fimmu.2024.1408928 (PMC11257869; doi:10.3389/fimmu.2024.1408928)
Supplement: Supplementary file 1 [file DataSheet_1.docx]

**First-Line treatments for extensive-stage small-cell lung cancer with immune checkpoint inhibitors plus chemotherapy: a China-based cost-effectiveness analysis**

**Table S1. First-line treatment strategies compared in the model**

**Table S2. AIC and BIC statistics for survival fitting of first-line chemotherapy**

**Table S3. Model inputs regarding transition probabilities estimation**

**Table S4. Derivation of AEs-related management costs**

**Table S5. Derivation of AEs-related utility decrements**

**Table S6. The calculation of grades 3/4 AEs-induced costs and utilities for each first-line treatment**

**Table S7. Model inputs regarding costs and health state utilities** **estimation**

**Table S8. Subgroup-level and stepwise ICERs comparison of first-line treatment strategies**

**Figure S1. KM curves and modeled curves for the OS of first-line chemotherapy.** KM, Kaplan-Meier; OS, overall survival.

**Figure S2. KM curves and modeled curves for the PFS of first-line chemotherapy.** KM, Kaplan-Meier; PFS, progression-free survival.

**Figure S3. One-way sensitivity analysis for HR of OS for serplulimab plus chemotherapy relative to chemotherapy.** ICER, incremental cost-effectiveness ratio; HR, hazard ratio; OS, overall survival.

**Figure S4.** **One-way sensitivity analysis for patients’ mean weight.** ICER, incremental cost-effectiveness ratio.

**Figure S5.** **One-way sensitivity analysis for the cost of serplulimab per 4.5mg/kg.** ICER, incremental cost-effectiveness ratio.

**Figure S6. Probabilistic sensitivity analyses results for male patients with ES-SCLC.** WTP, willingness-to-pay; ES-SCLC, extensive-stage small cell lung cancer.

**Figure S7. Probabilistic sensitivity analyses results for female patients with ES-SCLC.** WTP, willingness-to-pay; ES-SCLC, extensive-stage small cell lung cancer.

**Figure S8. Probabilistic sensitivity analyses results for age≥65 patients with ES-SCLC.** WTP, willingness-to-pay; ES-SCLC, extensive-stage small cell lung cancer.

**Figure S9. Probabilistic sensitivity analyses results for age<65 patients with ES-SCLC.** WTP, willingness-to-pay; ES-SCLC, extensive-stage small cell lung cancer.

**Figure S10. Probabilistic sensitivity analyses results for ES-SCLC patients with ECOG performance status score of 0.** WTP, willingness-to-pay; ES-SCLC, extensive-stage small cell lung cancer; ECOG, Eastern Cooperative Oncology Group.

**Figure S11. Probabilistic sensitivity analyses results for ES-SCLC patients with ECOG performance status score of 1.** WTP, willingness-to-pay; ES-SCLC, extensive-stage small cell lung cancer; ECOG, Eastern Cooperative Oncology Group.

**Table S1. First-line treatment strategies compared in the model**

| **Strategies** | **Treatment dosage** | **Administration schedule** |
| --- | --- | --- |
| Chemotherapy^a[1]^ | Etoposide, 100mg/m^2^ | On days 1,2 and 3 of each cycle for up to 4 cycles |
|  | Carboplatin, AUC 5.0 mg/ml/min | On day 1 of each cycle for up to 4 cycles |
| Atezolizumab+  Chemotherapy^a[2]^ | Atezolizumab, 1200mg | On day 1 of each cycle |
|  | Chemotherapy is the same as above | |
| Durvalumab^b^+  Chemotherapy^a[3]^ | Durvalumab, 1500 mg; 1125mg | On day 1 of each cycle for the first 4 cycles; On day 1 of each cycle for subsequent cycles |
|  | Chemotherapy is the same as above | |
| Durvalumab+  Tremelimumab+  Chemotherapy^a[4]^ | Durvalumab, 1500 mg; 1125mg | On day 1 of each cycle for the first 4 cycles; On day 1 of each cycle for subsequent cycles |
|  | Tremelimumab; 75mg | On day 1 of each cycle for the first 4 cycles |
|  | Chemotherapy is the same as above | |
| Serplulimab+  Chemotherapy^a[5]^ | Serplulimab, 4.5mg/kg | On day 1 of each cycle |
|  | Chemotherapy is the same as above | |
| Adebrelimab+  Chemotherapy^a[1]^ | Adebrelimab, 20mg/kg | On day 1 of each cycle |
|  | Chemotherapy is the same as above | |

Abbreviations: Chemo, AUC: area under curve

^a^This study modeled chemotherapy as etoposide plus carboplatin given that it is most commonly used chmotherapy in the first-line for extensive-stage small-cell lung cancer in China

^b^Durvalumab was adminstrated 1500 mg every 3 weeks for the first 4 cycles, followed by maintenance durvalumab 1500 mg every 4 weeks.

**Table S2. AIC and BIC statistics for survival fitting of first-line chemotherapy**

| **Parametric survival distribution** | **OS data** | | **PFS data** | |
| --- | --- | --- | --- | --- |
|  | **AIC** | **BIC** | **AIC** | **BIC** |
| Exponential | -71 | -67 | -45 | -42 |
| Weibull | -173 | -168 | -141 | -136 |
| Log-normal | -232 | -227 | -144 | -139 |
| Log-logistic^a^ | **-245** | **-239** | **-152** | **-147** |
| Gompertz | -244 | -237 | -146 | -139 |

Abbreviations: AIC, Akaike information criterion; BIC, Bayesian information criterion; OS, overall survival; PFS, progression-free survival.

^a^The log-logistic distribution was associated with the lowest AIC and BIC values, therefore it is considered to provide the best fit for first-line chemotherapy.

**Table S3. Model inputs regarding transition probabilities estimation**

| **Model inputs** | **Baseline Value** | **Range for DSA** | **Distribution for PSA** | **Source** |
| --- | --- | --- | --- | --- |
| OS for chemotherapy (Etoposide+carboplatin) | Log-logistic θ=0.0002938;κ=2.7910843 | Fixed | Fixed | Parametric survival analyses of CAPSTONE-1 data^[1]^ |
| PFS for chemotherapy (Etoposide+carboplatin) | Log-logistic θ=0.0001566;κ=4.1798674 | Fixed | Fixed |  |
| HR_os_ for atezolizumab+chemotherapy vs chemotherapy | 0.76 | 0.60-0.96 | LogNormal | NMA results^[6]^ |
| HR_os_ for durvalumab+chemotherapy vs chemotherapy | 0.75 | 0.62-0.91 | LogNormal |  |
| HR_os_ for durvalumab+tremelimumab+chemotherapy vs chemotherapy | 0.82 | 0.68-0.99 | LogNormal |  |
| HR_os_ for serplulimab+chemotherapy vs chemotherapy | 0.63 | 0.49-0.81 | LogNormal |  |
| HR_os_ for adebrelimab+chemotherapy vs chemotherapy | 0.72 | 0.58-0.90 | LogNormal |  |
| HR_PFS_ for atezolizumab+chemotherapy vs chemotherapy | 0.77 | 0.63-0.95 | LogNormal |  |
| HR_PFS_ for durvalumab+chemotherapy vs chemotherapy | 0.80 | 0.66-0.96 | LogNormal |  |
| HR_PFS_ for durvalumab+tremelimumab+chemotherapy vs chemotherapy | 0.84 | 0.70-1.01 | LogNormal |  |
| HR_PFS_ for serplulimab+chemotherapy vs chemotherapy | 0.48 | 0.39-0.60 | LogNormal |  |
| HR_PFS_ for adebrelimab+chemotherapy vs chemotherapy | 0.67 | 0.54-0.83 | LogNormal |  |
| HR_os_ (male) for atezolizumab+chemotherapy vs chemotherapy | 0.83 | 0.63-1.10 | LogNormal |  |
| HR_os_ (male) for durvalumab+chemotherapy vs chemotherapy | 0.79 | 0.63-0.99 | LogNormal |  |
| HR_os_ (male) for durvalumab+tremelimumab+chemotherapy vs chemotherapy | 0.83 | 0.67-1.04 | LogNormal |  |
| HR_os_ (male) for serplulimab+chemotherapy vs chemotherapy | 0.64 | 0.48-0.85 | LogNormal |  |
| HR_os_ (male) for adebrelimab+chemotherapy vs chemotherapy | 0.72 | 0.57-0.91 | LogNormal |  |
| HR_os_ (female) for atezolizumab+chemotherapy vs chemotherapy | 0.64 | 0.43-0.95 | LogNormal |  |
| HR_os_ (female) for durvalumab+chemotherapy vs chemotherapy | 0.65 | 0.45-0.93 | LogNormal |  |
| HR_os_ (female) for durvalumab+tremelimumab+chemotherapy vs chemotherapy | 0.76 | 0.52-1.10 | LogNormal |  |
| HR_os_ (female) for serplulimab+chemotherapy vs chemotherapy | 0.57 | 0.30-1.07 | LogNormal |  |
| HR_os_ (female) for adebrelimab+chemotherapy vs chemotherapy | 0.62 | 0.37-1.04 | LogNormal |  |
| HR_os_ (age≥ 65) for atezolizumab+chemotherapy vs chemotherapy | 0.59 | 0.42-0.82 | LogNormal |  |
| HR_os_ (age≥ 65) for durvalumab+chemotherapy vs chemotherapy | 0.84 | 0.62-1.13 | LogNormal |  |
| HR_os_ (age≥ 65) for durvalumab+tremelimumab+chemotherapy vs chemotherapy | 0.92 | 0.69-1.22 | LogNormal |  |
| HR_os_ (age≥ 65) for serplulimab+chemotherapy vs chemotherapy | 0.60 | 0.40-0.90 | LogNormal |  |
| HR_os_ (age≥ 65) for adebrelimab+chemotherapy vs chemotherapy | 0.70 | 0.48-1.01 | LogNormal |  |
| HR_os_ (age＜65) for atezolizumab+chemotherapy vs chemotherapy | 0.94 | 0.69-1.29 | LogNormal |  |
| HR_os_ (age＜65) for durvalumab+chemotherapy vs chemotherapy | 0.72 | 0.57-0.92 | LogNormal |  |
| HR_os_ (age＜65) for durvalumab+tremelimumab+chemotherapy vs chemotherapy | 0.76 | 0.59-0.97 | LogNormal |  |
| HR_os_ (age＜65) for serplulimab+chemotherapy vs chemotherapy | 0.62 | 0.45-0.86 | LogNormal |  |
| HR_os_ (age＜65) for adebrelimab+chemotherapy vs chemotherapy | 0.71 | 0.54-0.93 | LogNormal |  |
| HR_os_ (ECOG PS of 0) for atezolizumab+chemotherapy vs chemotherapy | 0.73 | 0.48-1.10 | LogNormal |  |
| HR_os_ (ECOG PS of 0) for durvalumab+chemotherapy vs chemotherapy | 0.77 | 0.56-1.07 | LogNormal |  |
| HR_os_ (ECOG PS of 0) for durvalumab+tremelimumab+chemotherapy vs chemotherapy | 0.79 | 0.58-1.09 | LogNormal |  |
| HR_os_ (ECOG PS of 0) for serplulimab+chemotherapy vs chemotherapy | 0.44 | 0.23-0.84 | LogNormal |  |
| HR_os_ (ECOG PS of 0) for adebrelimab+chemotherapy vs chemotherapy | 0.83 | 0.46-1.51 | LogNormal |  |
| HR_os_ (ECOG PS of 1) for atezolizumab+chemotherapy vs chemotherapy | 0.78 | 0.60-1.02 | LogNormal |  |
| HR_os_ (ECOG PS of 1) for durvalumab+chemotherapy vs chemotherapy | 0.76 | 0.60-0.96 | LogNormal |  |
| HR_os_ (ECOG PS of 1) for durvalumab+tremelimumab+chemotherapy vs chemotherapy | 0.87 | 0.69-1.10 | LogNormal |  |
| HR_os_ (ECOG PS of 1) for serplulimab+chemotherapy vs chemotherapy | 0.65 | 0.49-0.86 | LogNormal |  |
| HR_os_ (ECOG PS of 1) for adebrelimab+chemotherapy vs chemotherapy | 0.69 | 0.55-0.87 | LogNormal |  |

Abbreviations: DSA, deterministic sensitivity analyses; PSA, probabilistic sensitivity analyses; OS, overall survival; PFS, progression-free survival; NMA, network meta-analysis; HR, hazard ratio; OS ECOG, Eastern Cooperative Oncology Group; PS, performance status.

**Table S4. Derivation of AEs-related management costs**

| **AEs** | **Medication** | | **Examination** | | **Hospitalization** | | **Cost per event ($)** | **Reference** |
| --- | --- | --- | --- | --- | --- | --- | --- | --- |
|  | **Drugs** | **Cost^a^ ($)** | **Items** | **Cost^a^ ($)** | **Duration** | **Cost^a^ ($)** |  |  |
| Neutrophil count decreased | Leukopoietin | 31.47 | Routine blood test | 8.09 | 7.00 | 49.67 | 89.23 | Expert consensus^[7]^ |
| White blood cell count decreased | Leukopoietin | 31.47 | Routine blood test | 8.09 | 7.00 | 49.67 | 89.23 | Expert consensus^[7]^ |
| Platelet count decreased | Thrombopoietin | 196.27 | Routine blood test | 8.09 | 7.00 | 49.67 | 254.02 | Expert consensus^[8]^ |
| ALT increased | Hepatinica | 10.90 | Liver function tests; abdominal CT | 103.88 | 5.00 | 35.48 | 150.25 | Expert consensus^[8]^ |
| AST increased | Hepatinica | 10.90 | Liver function tests; abdominal CT | 103.88 | 5.00 | 35.48 | 150.25 | Expert consensus^[8]^ |
| γ-glutamyltransferase increased | Hepatinica | 10.90 | Liver function tests; abdominal CT | 103.88 | 5.00 | 35.48 | 150.25 | Expert consensus^[8]^ |
| Anaemia | Erythropoietin | 3.53 | Routine blood test | 8.09 | 5.00 | 35.48 | 47.09 | Expert consensus^[9]^ |
| Vomiting | Antiemetic | 12.54 | Electrolyte examination | 4.12 | 5.00 | 35.48 | 52.13 | Expert consensus^[10]^ |
| Decreased appetite | lutin | 1.11 | Electrolyte examination | 4.12 | 5.00 | 35.48 | 40.70 | Expert consensus^[11]^ |
| Asthenia | Blood tonics | 46.83 | Routine blood test | 5.39 | 7.00 | 49.67 | 101.89 | Local oncologists |
| Hyponatraemia | Electrolyte supplements | 0.29 | Electrolyte examination | 6.17 | 5.00 | 35.48 | 41.94 | Local oncologists |
| Hypertension | Depressor | 4.17 | Electrocardiogram; Echocardiography; Myocardial enzymes | 69.11 | 7.00 | 49.67 | 122.95 | Expert consensus^[12]^ |
| Pneumonia | Antibiotics; Cough medicine; Glucocorticoids | 53.58 | Chest CT | 110.69 | 10.00 | 70.96 | 235.23 | Expert consensus^[13]^ |
| Lymphocyte count decreased^b^ | / | / | / | / | / | / | / | Local oncologists’ opinion |
| Febrile neutropenia | Leukopoietin; Antibiotics; Glucocorticoids | 260.78 | Rutine blood test; Chest CT | 123.46 | 10.00 | 70.96 | 455.20 | Expert consensus^[7]^ |
| Myelosuppression | Leukopoietin | 73.43 | Routine blood test | 8.09 | 7.00 | 49.67 | 131.19 | Expert consensus^[14]^ |

Abbreviations: AEs, adverse events; ALT, alanine aminotransferase; AST, aspartate aminotransferase; CT, Computed Tomography. ^a^These costs were estimated using data from local comprehensive hospitals.

^b^Lymphocyte count decreased does not require additional treatment, according to local oncologists’ opinion.

**Table S5 Derivation of AEs-Related Utility Decrements**

| **AEs** | **Disutilities** | **Source** | **Duration (Days)** | **Source** | **Disutilities Decrement** |
| --- | --- | --- | --- | --- | --- |
| Neutrophil count decreased | 0.08973 | ICER^[15]^ | 16.80 | Delanoy N, et al^[16]^ | 0.00413 |
| White blood cell count decreased | 0.08973 |  | 16.80 | Delanoy N, et al^[16]^ | 0.00413 |
| Platelet count decreased | 0.08973 |  | 30.80 | Delanoy N, et al^[16]^ | 0.00757 |
| ALT increased | 0.04680 |  | 56.70 | Borghaei H, et al^[17]^ | 0.00727 |
| AST increased | 0.04680 |  | 56.70 | Borghaei H, et al^[17]^ | 0.00727 |
| γ-glutamyltransferase increased | 0.04680 |  | 56.70 | Borghaei H, et al^[17]^ | 0.00727 |
| Anaemia | 0.08973 |  | 42.00 | Delanoy N, et al^[16]^ | 0.01033 |
| Vomiting | 0.04802 |  | 14.00 | Borghaei H, et al^[17]^ | 0.00184 |
| Decreased appetite | 0.07346 |  | 14.00 | Borghaei H, et al^[17]^ | 0.00282 |
| Asthenia | 0.07346 |  | 5.00 | Msaouel P, et al^[18]^ | 0.00101 |
| Hyponatraemia | 0.08973 |  | 55.30 | Brahmer J, et al^[19]^ | 0.01359 |
| Hypertension | 0.08973 |  | 28.00 | Local oncologists | 0.00688 |
| Pneumonia | 0.07346 |  | 41.30 | Borghaei H, et al^[17]^ | 0.00831 |
| Lymphocyte count decreased | 0.08973 |  | 16.80 | Delanoy N, et al^[16]^ | 0.00413 |
| Febrile neutropenia | 0.09002 |  | 56.00 | Delanoy N, et al^[16]^ | 0.01381 |
| Myelosuppression | 0.09002 |  | 56.00 | Delanoy N, et al^[16]^ | 0.01381 |

Abbreviations: AEs, adverse events; ALT, alanine aminotransferase; AST, aspartate aminotransferase; ICER, Institute for clinical and economic review.

**Table S6. The calculation of grades 3/4 AEs-induced costs and utilities for each first-line treatment**

| AEs | Proportion (%) | HRs^[6]^ | | | | | Cost per event($) | Disutility |
| --- | --- | --- | --- | --- | --- | --- | --- | --- |
|  |  | Atezolizumab+chemotherapy vs chemotherapy | Durvalumab+ chemotherapy vs chemotherapy | Durvalumab+ Tremelimumab+  chemotherapy vs chemotherapy | Serplulimab+ chemotherapy vs chemotherapy | Adebrelimab+ chemotherapy vs chemotherapy |  |  |
| Neutrophil count decreased | 75.43% | 1.04 (0.68, 1.60) | 0.98 (0.68, 1.40) | 1.51 (1.04, 2.19) | 1.17 (0.75, 1.81) | 1.06 (0.63, 1.78) | 89.23 | 0.00413 |
| White blood cell count decreased | 37.93% |  |  |  |  |  | 89.23 | 0.00413 |
| Platelet count decreased | 33.62% |  |  |  |  |  | 254.02 | 0.00757 |
| ALT increased | 1.72% |  |  |  |  |  | 150.25 | 0.00727 |
| AST increased | 1.72% |  |  |  |  |  | 150.25 | 0.00727 |
| γ-glutamyltransferase increased | 0.43% |  |  |  |  |  | 150.25 | 0.00727 |
| Anaemia | 28.45% |  |  |  |  |  | 47.09 | 0.01033 |
| Vomiting | 0.43% |  |  |  |  |  | 52.13 | 0.00184 |
| Decreased appetite | 0.86% |  |  |  |  |  | 40.70 | 0.00282 |
| Asthenia | 0.43% |  |  |  |  |  | 101.89 | 0.00101 |
| Hyponatraemia | 2.59% |  |  |  |  |  | 41.94 | 0.01359 |
| Hypertension | 0.86% |  |  |  |  |  | 122.95 | 0.00688 |
| Pneumonia | 0.43% |  |  |  |  |  | 235.23 | 0.00831 |
| Lymphocyte count decreased | 1.72% |  |  |  |  |  | / | 0.00413 |
| Febrile neutropenia | 0.86% |  |  |  |  |  | 455.20 | 0.01381 |
| Myelosuppression | 1.29% |  |  |  |  |  | 131.19 | 0.01381 |
| Estimated AEs Costs and disutilities | | | | | | |  |  |
| AEs cost for first-line chemotherapy, $ | | | | | | | 215.58 |  |
| AEs cost for first-line atezolizumab+chemotherapy, $ | | | | | | | 224.20 |  |
| AEs cost for first-line durvalumab+chemotherapy, $ | | | | | | | 211.27 |  |
| AEs cost for first-line durvalumab+tremelimumab+chemotherapy, $ | | | | | | | 325.52 |  |
| AEs cost for first-line serplulimab+chemotherapy, $ | | | | | | | 252.23 |  |
| AEs cost for first-line adebrelimab+chemotherapy, $ | | | | | | | 228.51 |  |
| AEs disutility for first-line chemotherapy, $ | | | | | | | | 0.01130 |
| AEs disutility for first-line atezolizumab+chemotherapy, $ | | | | | | | | 0.01175 |
| AEs disutility for first-line durvalumab+chemotherapy, $ | | | | | | | | 0.01107 |
| AEs disutility for first-line durvalumab+tremelimumab+chemotherapy, $ | | | | | | | | 0.01706 |
| AEs disutility for first-line serplulimab+chemotherapy, $ | | | | | | | | 0.01322 |
| AEs disutility for first-line adebrelimab+chemotherapy, $ | | | | | | | | 0.01198 |

Abbreviations: HRs, hazard ratios; AEs, adverse events; ALT, alanine aminotransferase; AST, aspartate aminotransferase.

**Table S7. Model inputs regarding costs and health state utilities** **estimation**

| **Model inputs** | **Baseline Value** | **Range for DSA** | **Distribution for PSA** | **Source** |
| --- | --- | --- | --- | --- |
| **Costs (US$)** | | | | |
| Tremelimumab per 75mg | 7215.70 | 5411.78-9019.63 | Gamma | Hong Kong Jimin Pharmaceutical^[20]^ |
| Atezolizumab per 1200mg | 4654.66 | 3491.00-5818.33 | Gamma | National Health Industry Data Platform^[21]^ |
| Durvalumab per 1500mg | 7700.29 | 5775.21-9625.36 | Gamma |  |
| Adebrelimab per 20mg/kg | 44.94 | 33.70-56.17 | Gamma |  |
| Serplulimab per 4.5mg/kg | 35.68 | 26.76-44.61 | Gamma |  |
| Etoposide per 300mg/m^2^ | 3.32 | 2.49-4.15 | Gamma |  |
| Carboplatin per 5.0 mg/ml/min | 1.12 | 0.84-1.40 | Gamma |  |
| Subsequent anticancer therapies per cycle | 854.05 | 640.54-1067.56 | Gamma | Luo X, et al^[22]^ |
| Routine follow-up per cycle | 55.60 | 41.70-69.50 | Gamma |  |
| BSC per cycle | 337.50 | 253.13-421.88 | Gamma |  |
| Palliative care per cycle | 2627.80 | 1970.85-3284.75 | Gamma |  |
| AEs cost for chemotherapy | 215.58 | 161.68-269.47 | Gamma | Supplementary Table S6 |
| AEs cost for atezolizumab+chemotherapy | 224.20 | 168.15-280.25 | Gamma |  |
| AEs cost for durvalumab+chemotherapy | 211.27 | 158.45-264.08 | Gamma |  |
| AEs cost for durvalumab+tremelimumab+chemotherapy | 325.52 | 244.14-406.91 | Gamma |  |
| AEs cost for serplulimab+chemotherapy | 252.23 | 189.17-315.28 | Gamma |  |
| AEs cost for adebrelimab+chemotherapy | 228.51 | 171.39-285.64 | Gamma |  |
| **Health state utilities** | | | | |
| PFD health state | 0.85600 | 0.64200-1.00000 | Beta | Shen Y, et al^[23]^ |
| PD health state | 0.76800 | 0.57600-0.96000 | Beta |  |
| AEs disutility for chemotherapy | 0.01130 | 0.00847-0.01412 | Beta | Supplementary Table S6 |
| AEs disutility for atezolizumab+chemotherapy | 0.01175 | 0.00881-0.01469 | Beta |  |
| AEs disutility for durvalumab+chemotherapy | 0.01107 | 0.00830-0.01384 | Beta |  |
| AEs disutility for durvalumab+tremelimumab+chemotherapy | 0.01706 | 0.01280-0.02133 | Beta |  |
| AEs disutility for serplulimab+chemotherapy | 0.01322 | 0.00991-0.01652 | Beta |  |
| AEs disutility for adebrelimab+chemotherapy | 0.01198 | 0.00898-0.01497 | Beta |  |
| **Other** |  |  |  |  |
| Discount rate | 0.05 | 0.00-0.08 | Fixed | China Guidelines for Pharmacoeconomic Evaluations^[24]^ |
| Body surface area(m^2^) | 1.72 | 1.29-2.15 | Normal | Luo X, et al^[22]^ |
| Creatinine clearance rate (ml/min) | 70.00 | 52.50-87.50 | Normal |  |
| Mean weight (kg) | 67.59 | 50.69-84.48 | Normal |  |
| Male weight (kg) | 69.60 | 52.20-87.00 | Normal | National Health Commission of the People's Republic of China^[25]^ |
| Female weight (kg) | 59.00 | 44.25-73.75 | Normal |  |

Abbreviations: DSA, deterministic sensitivity analyses; PSA, probabilistic sensitivity analyses; BSC, best supportive care; AEs, advent events; PFD, progression-free disease, PD, progressed disease.

**Table S8. Subgroup-level and stepwise ICERs comparison of first-line treatment strategies**

| **Male subgroup** | | | | | | |
| --- | --- | --- | --- | --- | --- | --- |
| **ICERs ( vs chemotherapy)** | | | | | | |
| **Strategy** | **Costs (US$)** | **QALYs** | **Incremental costs** | **Incremental QALYs** | | **ICER ($/QALY)** |
| Chemotherapy | 21,409.67 | 1.02657 | NA | NA | | NA |
| Serplulimab+Chemotherapy | 59,945.79 | 1.49201 | 38,536.12 | 0.46544 | | 82,795.84 |
| Adebrelimab+Chemotherapy | 60,235.07 | 1.33257 | 38,825.40 | 0.30600 | | 126,879.72 |
| Atezolizumab+Chemotherapy | 70,869.78 | 1.18374 | 49,460.11 | 0.15717 | | 314,689.17 |
| Durvalumab+Chemotherapy | 89,077.60 | 1.22955 | 67,667.92 | 0.20298 | | 333,375.39 |
| Durvalumab+Tremelimumab+Chemotherapy | 155,637.76 | 1.18097 | 134,228.09 | 0.15439 | | 869,397.08 |
| **Stepwise ICERs comparison** | | | | | | |
| **Strategy^a^** | **Costs (US$)** | **QALYs** | **Incremental costs^b^** | **Incremental QALYs^b^** | | **ICER ($/QALY)^c^** |
| Chemotherapy | 21,409.67 | 1.02657 | NA | NA | | NA |
| Serplulimab+Chemotherapy | 59,945.79 | 1.49201 | 38,536.12 | 0.46544 | | 82,795.84 |
| Adebrelimab+Chemotherapy | 60,235.07 | 1.33257 | 289.28 | -0.15943 **(D)** | | -1,814.43 |
| Atezolizumab+Chemotherapy | 70,869.78 | 1.18374 | 10,634.71 | -0.14883 **(D)** | | -71,455.31 |
| Durvalumab+Chemotherapy | 89,077.60 | 1.22955 | 18,207.81 | 0.04581 | | 397,491.08 |
| Durvalumab+Tremelimumab+Chemotherapy | 155,637.76 | 1.18097 | 66,560.17 | -0.04859 **(D)** | | -1,369,945.48 |
| Excluding dominated strategies: | | | | | | |
| Chemotherapy | 21,409.67 | 1.02657 | NA | NA | | NA |
| Serplulimab+Chemotherapy | 59,945.79 | 1.49201 | 38,536.12 | 0.46544 | | 82,795.84 |
| Durvalumab+Chemotherapy | 89,077.60 | 1.22955 | 29,131.81 | -0.26246 **(D)** | | -110,996.39 |
| Excluding dominated strategies: | | | | | | |
| Chemotherapy | 21,409.67 | 1.02657 | NA | NA | | NA |
| Serplulimab+Chemotherapy | 59,945.79 | 1.49201 | 38,536.12 | 0.46544 | | 82,795.84 |
| **Female subgroup** | | | | | | |
| **ICERs ( vs chemotherapy)** | | | | | | |
| **Strategy** | **Costs (US$)** | **QALYs** | **Incremental costs** | **Incremental QALYs** | **ICER ($/QALY)** | |
| Chemotherapy | 21,409.67 | 1.02657 | NA | NA | NA | |
| Serplulimab+Chemotherapy | 64,451.47 | 1.65578 | 43,041.79 | 0.62921 | 68,406.41 | |
| Adebrelimab+Chemotherapy | 65,332.13 | 1.51781 | 43,922.45 | 0.49124 | 89,411.53 | |
| Atezolizumab+Chemotherapy | 78,766.73 | 1.47068 | 57,357.06 | 0.44410 | 129,152.25 | |
| Durvalumab+Chemotherapy | 95,131.17 | 1.44949 | 73,721.49 | 0.42291 | 174,317.91 | |
| Durvalumab+Tremelimumab+Chemotherapy | 158,005.67 | 1.26693 | 136,596.00 | 0.24036 | 568,302.83 | |
| **Stepwise ICERs comparison** | | | | | | |
| **Strategy^a^** | **Costs (US$)** | **QALYs** | **Incremental costs^b^** | **Incremental QALYs^b^** | **ICER ($/QALY)^c^** | |
| Chemotherapy | 21,409.67 | 1.02657 | NA | NA | NA | |
| Serplulimab+Chemotherapy | 64,451.47 | 1.65578 | 43,041.79 | 0.62921 | 68,406.41 | |
| Adebrelimab+Chemotherapy | 65,332.13 | 1.51781 | 880.66 | -0.13797 **(D)** | -6,383.11 | |
| Atezolizumab+Chemotherapy | 78,766.73 | 1.47068 | 13,434.60 | -0.04714 **(D)** | -285,023.46 | |
| Durvalumab+Chemotherapy | 95,131.17 | 1.44949 | 16,364.44 | -0.02119 **(D)** | -772,266.46 | |
| Durvalumab+Tremelimumab+Chemotherapy | 158,005.67 | 1.26693 | 62,874.50 | -0.18256 **(D)** | -344,411.57 | |
| Excluding dominated strategies: | | | | | | |
| Chemotherapy | 21,409.67 | 1.02657 | NA | NA | NA | |
| Serplulimab+Chemotherapy | 64,451.47 | 1.65578 | 43,041.79 | 0.62921 | 68,406.41 | |
| **Age≥ 65 subgroup** | | | | | | |
| **ICERs ( vs chemotherapy)** | | | | | | |
| **Strategy** | **Costs (US$)** | **QALYs** | **Incremental costs** | **Incremental QALYs** | **ICER ($/QALY)** | |
| Chemotherapy | 21,409.67 | 1.02657 | NA | NA | NA | |
| Adebrelimab+Chemotherapy | 61,118.31 | 1.36467 | 39,708.64 | 0.33810 | 117,447.25 | |
| Serplulimab+Chemotherapy | 62,373.65 | 1.58025 | 40,963.97 | 0.55368 | 73,984.99 | |
| Atezolizumab+Chemotherapy | 81,860.95 | 1.58313 | 60,451.27 | 0.55656 | 108,615.96 | |
| Durvalumab+Chemotherapy | 87,486.93 | 1.17177 | 66,077.26 | 0.14520 | 455,075.96 | |
| Durvalumab+Tremelimumab+Chemotherapy | 153,214.63 | 1.09302 | 131,804.96 | 0.06645 | 1,983,528.56 | |
| **Stepwise ICERs comparison** | | | | | | |
| **Strategy^a^** | **Costs (US$)** | **QALYs** | **Incremental costs^b^** | **Incremental QALYs^b^** | **ICER ($/QALY)^c^** | |
| Chemotherapy | 21,409.67 | 1.02657 | NA | NA | NA | |
| Adebrelimab+Chemotherapy | 61,118.31 | 1.36467 | 39,708.64 | 0.33810 | 117,447.25 **(ED)** | |
| Serplulimab+Chemotherapy | 62,373.65 | 1.58025 | 1,255.34 | 0.21558 | 5,823.02 | |
| Atezolizumab+Chemotherapy | 81,860.95 | 1.58313 | 19,487.30 | 0.00288 | 6,765,932.52 | |
| Durvalumab+Chemotherapy | 87,486.93 | 1.17177 | 5,625.99 | -0.41136 **(D)** | -13,676.58 | |
| Durvalumab+Tremelimumab+Chemotherapy | 153,214.63 | 1.09302 | 65,727.70 | -0.07875 **(D)** | -834,629.37 | |
| Excluding dominated and extended dominated strategies: | | | | | | |
| Chemotherapy | 21,409.67 | 1.02657 | NA | NA | NA | |
| Serplulimab+Chemotherapy | 62,373.65 | 1.58025 | 40,963.97 | 0.55368 | 73,984.99 | |
| Atezolizumab+Chemotherapy | 81,860.95 | 1.58313 | 19,487.30 | 0.00288 | 6,765,932.52 | |
| **Age＜65 subgroup** | | | | | | |
| **ICERs ( vs chemotherapy)** | | | | | | |
| **Strategy** | **Costs (US$)** | **QALYs** | **Incremental costs** | **Incremental QALYs** | **ICER ($/QALY)** | |
| Chemotherapy | 21,409.67 | 1.02657 | NA | NA | NA | |
| Adebrelimab+Chemotherapy | 60,669.32 | 1.34835 | 39,259.64 | 0.32178 | 122,007.14 | |
| Serplulimab+Chemotherapy | 61,114.69 | 1.53449 | 39,705.01 | 0.50792 | 78,171.81 | |
| Atezolizumab+Chemotherapy | 67,985.07 | 1.07896 | 46,575.40 | 0.05239 | 889,059.07 | |
| Durvalumab+Chemotherapy | 91,755.74 | 1.32684 | 70,346.07 | 0.30027 | 234,276.00 | |
| Durvalumab+Tremelimumab+Chemotherapy | 158,005.67 | 1.26693 | 136,596.00 | 0.24036 | 568,302.83 | |
| **Stepwise ICERs comparison** | | | | | | |
| **Strategy^a^** | **Costs (US$)** | **QALYs** | **Incremental costs^b^** | **Incremental QALYs^b^** | **ICER ($/QALY)^c^** | |
| Chemotherapy | 21,409.67 | 1.02657 | NA | NA | NA | |
| Adebrelimab+Chemotherapy | 60,669.32 | 1.34835 | 39,259.64 | 0.32178 | 122,007.14 **(ED)** | |
| Serplulimab+Chemotherapy | 61,114.69 | 1.53449 | 445.37 | 0.18614 | 2,392.69 | |
| Atezolizumab+Chemotherapy | 67,985.07 | 1.07896 | 6,870.38 | -0.45553 **(D)** | -15,082.09 | |
| Durvalumab+Chemotherapy | 91,755.74 | 1.32684 | 23,770.67 | 0.24788 | 95,894.80 | |
| Durvalumab+Tremelimumab+Chemotherapy | 158,005.67 | 1.26693 | 66,249.93 | -0.05991 **(D)** | -1,105,782.02 | |
| Excluding dominated and extended dominated strategies: | | | | | | |
| Chemotherapy | 21,409.67 | 1.02657 | NA | NA | NA | |
| Serplulimab+Chemotherapy | 61,114.69 | 1.53449 | 39,705.01 | 0.50792 | 78,171.81 | |
| Durvalumab+Chemotherapy | 91,755.74 | 1.32684 | 30,641.05 | -0.20765 **(D)** | -147,561.21 | |
| Excluding dominated strategies: | | | | | | |
| Chemotherapy | 21,409.67 | 1.02657 | NA | NA | NA | |
| Serplulimab+Chemotherapy | 61,114.69 | 1.53449 | 39,705.01 | 0.50792 | 78,171.81 | |
| **ECOG PS of 0 subgroup** | | | | | | |
| **ICERs ( vs chemotherapy)** | | | | | | |
| **Strategy** | **Costs (US$)** | **QALYs** | **Incremental costs** | **Incremental QALYs** | **ICER ($/QALY)** | |
| Chemotherapy | 21,409.67 | 1.02657 | NA | NA | NA | |
| Adebrelimab+Chemotherapy | 56,267.03 | 1.18839 | 34,857.36 | 0.16182 | 215,405.81 | |
| Atezolizumab+Chemotherapy | 74,418.08 | 1.31266 | 53,008.41 | 0.28608 | 185,289.97 | |
| Serplulimab+Chemotherapy | 77,067.76 | 2.11448 | 55,658.09 | 1.08791 | 51,160.58 | |
| Durvalumab+Chemotherapy | 89,783.62 | 1.25520 | 68,373.94 | 0.22862 | 299,065.98 | |
| Durvalumab+Tremelimumab+Chemotherapy | 156,929.36 | 1.22785 | 135,519.68 | 0.20128 | 673,289.89 | |
| **Stepwise ICERs comparison** | | | | | | |
| **Strategy^a^** | **Costs (US$)** | **QALYs** | **Incremental costs^b^** | **Incremental QALYs^b^** | **ICER ($/QALY)^c^** | |
| Chemotherapy | 21,409.67 | 1.02657 | NA | NA | NA | |
| Adebrelimab+Chemotherapy | 56,267.03 | 1.18839 | 34,857.36 | 0.16182 | 215,405.81 | |
| Atezolizumab+Chemotherapy | 74,418.08 | 1.31266 | 18,151.05 | 0.12426 | 146,071.14 **(ED)** | |
| Serplulimab+Chemotherapy | 77,067.76 | 2.11448 | 2,649.68 | 0.80183 | 3,304.56 | |
| Durvalumab+Chemotherapy | 89,783.62 | 1.25520 | 12,715.86 | -0.85928 **(D)** | -14,798.19 | |
| Durvalumab+Tremelimumab+Chemotherapy | 156,929.36 | 1.22785 | 67,145.74 | -0.02735 **(D)** | -2,455,494.85 | |
| Excluding dominated and extended dominated strategies: | | | | | | |
| Chemotherapy | 21,409.67 | 1.02657 | NA | NA | NA | |
| Adebrelimab+Chemotherapy | 56,267.03 | 1.18839 | 34,857.36 | 0.16182 | 215,405.81**(ED)** | |
| Serplulimab+Chemotherapy | 77,067.76 | 2.11448 | 20,800.73 | 0.92609 | 22,460.86 | |
| Excluding extended dominated strategies: | | | | | | |
| Chemotherapy | 21,409.67 | 1.02657 | NA | NA | NA | |
| Serplulimab+Chemotherapy | 77,067.76 | 2.11448 | 55,658.09 | 1.08791 | 51,160.58 | |
| **ECOG PS of 1 subgroup** | | | | | | |
| **ICERs ( vs chemotherapy)** | | | | | | |
| **Strategy** | **Costs (US$)** | **QALYs** | **Incremental costs** | **Incremental QALYs** | **ICER ($/QALY)** | |
| Chemotherapy | 21,409.67 | 1.02657 | NA | NA | NA | |
| Serplulimab+Chemotherapy | 59,392.49 | 1.47190 | 37,982.82 | 0.44533 | 85,292.14 | |
| Adebrelimab+Chemotherapy | 61,582.74 | 1.38155 | 40,173.06 | 0.35497 | 113,171.55 | |
| Atezolizumab+Chemotherapy | 72,507.99 | 1.24326 | 51,098.32 | 0.21669 | 235,817.78 | |
| Durvalumab+Chemotherapy | 90,153.34 | 1.26863 | 68,743.67 | 0.24206 | 283,998.93 | |
| Durvalumab+Tremelimumab+Chemotherapy | 154,486.93 | 1.13919 | 133,077.26 | 0.11262 | 1,181,636.41 | |
| **Stepwise ICERs comparison** | | | | | | |
| **Strategy^a^** | **Costs (US$)** | **QALYs** | **Incremental costs^b^** | **Incremental QALYs^b^** | **ICER ($/QALY)^c^** | |
| Chemotherapy | 21,409.67 | 1.02657 | NA | NA | NA | |
| Serplulimab+Chemotherapy | 59,392.49 | 1.47190 | 37,982.82 | 0.44533 | 85,292.14 | |
| Adebrelimab+Chemotherapy | 61,582.74 | 1.38155 | 2,190.24 | -0.09035 **(D)** | -24,241.45 | |
| Atezolizumab+Chemotherapy | 72,507.99 | 1.24326 | 10,925.26 | -0.13829 **(D)** | -79,002.91 | |
| Durvalumab+Chemotherapy | 90,153.34 | 1.26863 | 17,645.35 | 0.02537 | 695,507.46 | |
| Durvalumab+Tremelimumab+Chemotherapy | 154,486.93 | 1.13919 | 64,333.59 | -0.12943 **(D)** | -497,034.24 | |
| Excluding dominated strategies: |  |  |  |  |  | |
| Chemotherapy | 21,409.67 | 1.02657 | NA | NA | NA | |
| Serplulimab+Chemotherapy | 59,392.49 | 1.47190 | 37,982.82 | 0.44533 | 85,292.14 | |
| Durvalumab+Chemotherapy | 90,153.34 | 1.26863 | 30,760.85 | -0.20327 **(D)** | -151,329.99 | |
| Excluding dominated strategies: |  |  |  |  |  | |
| Chemotherapy | 21,409.67 | 1.02657 | NA | NA | NA | |
| Serplulimab+Chemotherapy | 59,392.49 | 1.47190 | 37,982.82 | 0.44533 | 85,292.14 | |

Abbreviations: QALYs, quality-adjusted life-years; ICERs, incremental cost-effectiveness ratios; NA, not applicable; D, dominated strategy(a strategy is less effective and more costly than its previous alternative strategy). ED, extended dominated strategy (a strategy is less effective and less cost-effective than its next alternative strategy).

^a^For a stepwise ICER comparison, all competitive strategies are arranged in ascending order of cost.

^b^The increment costs and QALYs were calculated as the differences between the current strategy and its previous alternative.

^c^During each round of comparison, once dominated and extended dominated strategies are identified, they are excluded from the next round of comparison.

**Figure S1. KM curves and modeled curves for the OS of first-line chemotherapy.**

**
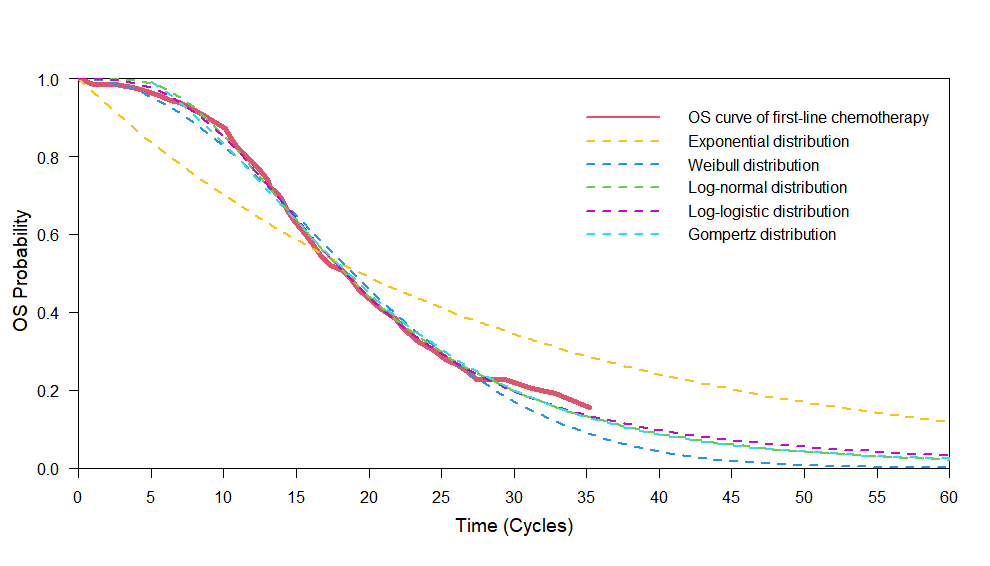
**

Abbreviations: KM, Kaplan-Meier; OS, overall survival.

**Figure S2. KM curves and modeled curves for the PFS of first-line chemotherapy.**


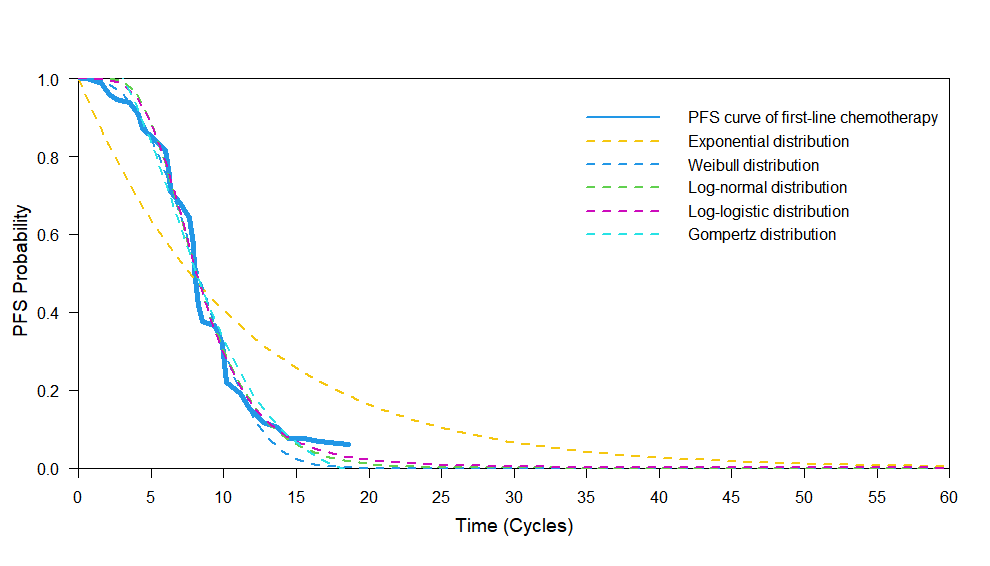


Abbreviations: KM, Kaplan-Meier; PFS, progression-free survival.

**Figure S3. One-way sensitivity analysis for HR of OS for serplulimab plus chemotherapy relative to chemotherapy.**

**
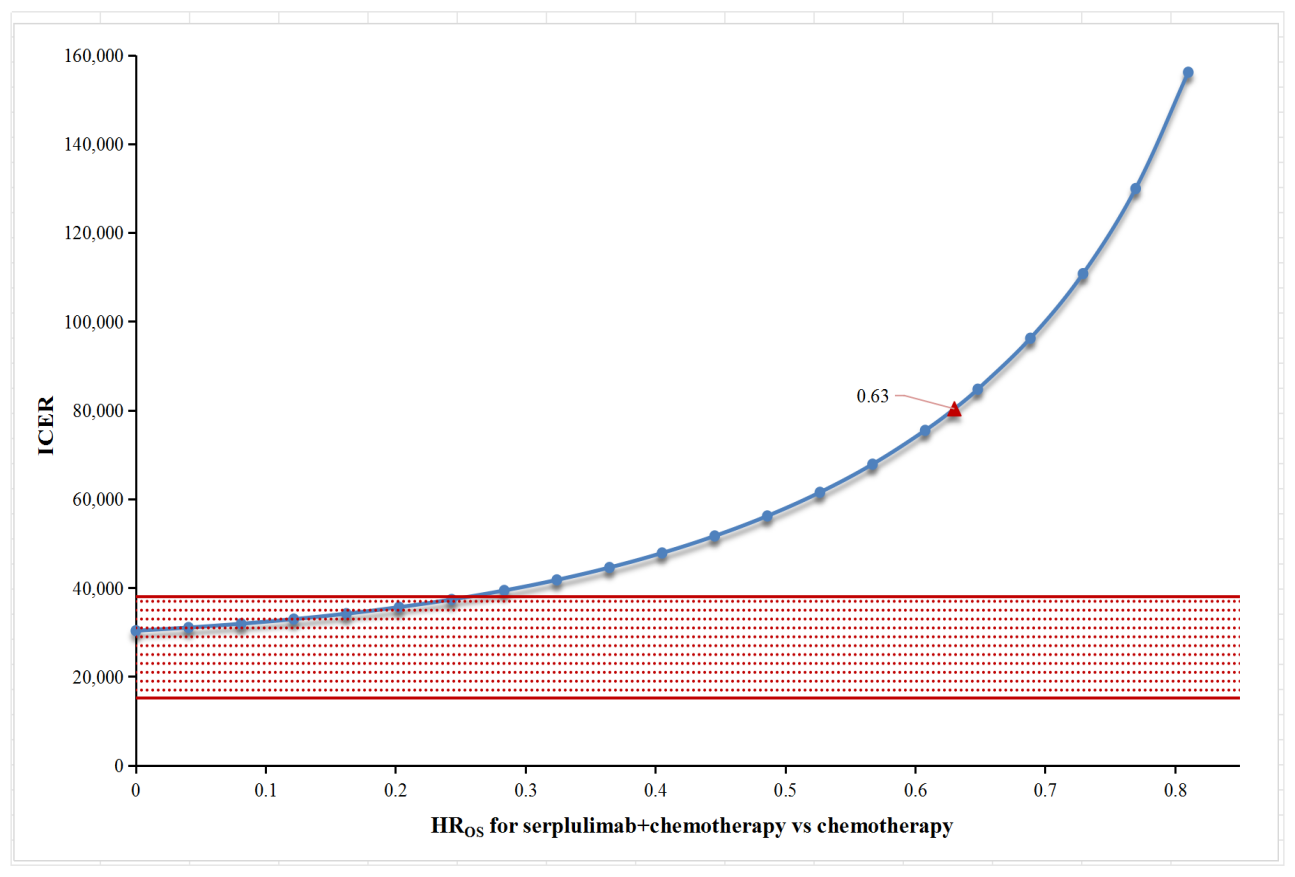
**

ICER, incremental cost-effectiveness ratio; HR, hazard ratio; OS, overall survival.

**Figure S4.** **One-way sensitivity analysis for patients’ mean weight.**


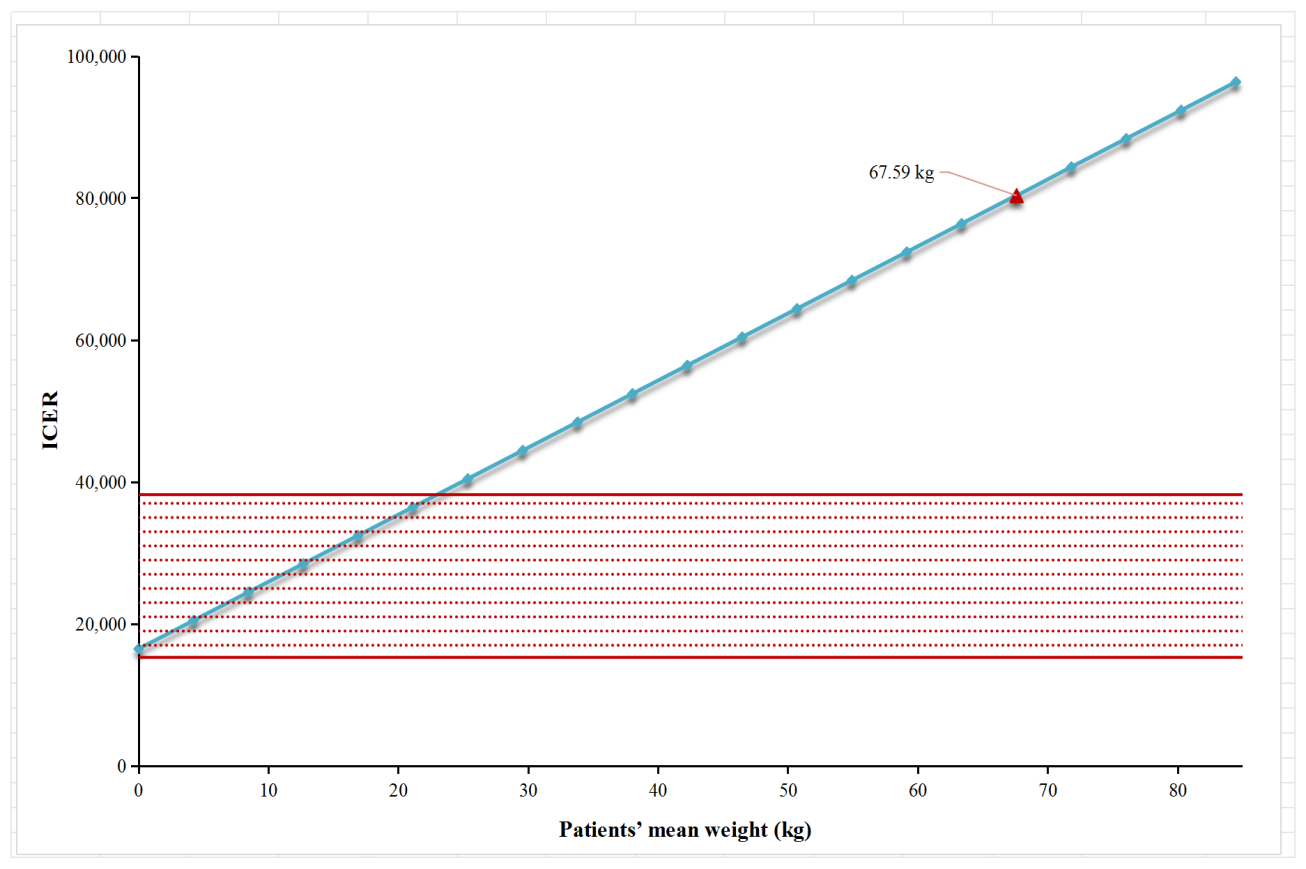


ICER, incremental cost-effectiveness ratio.

**Figure S5.** **One-way sensitivity analysis for the cost of serplulimab per 4.5mg/kg**


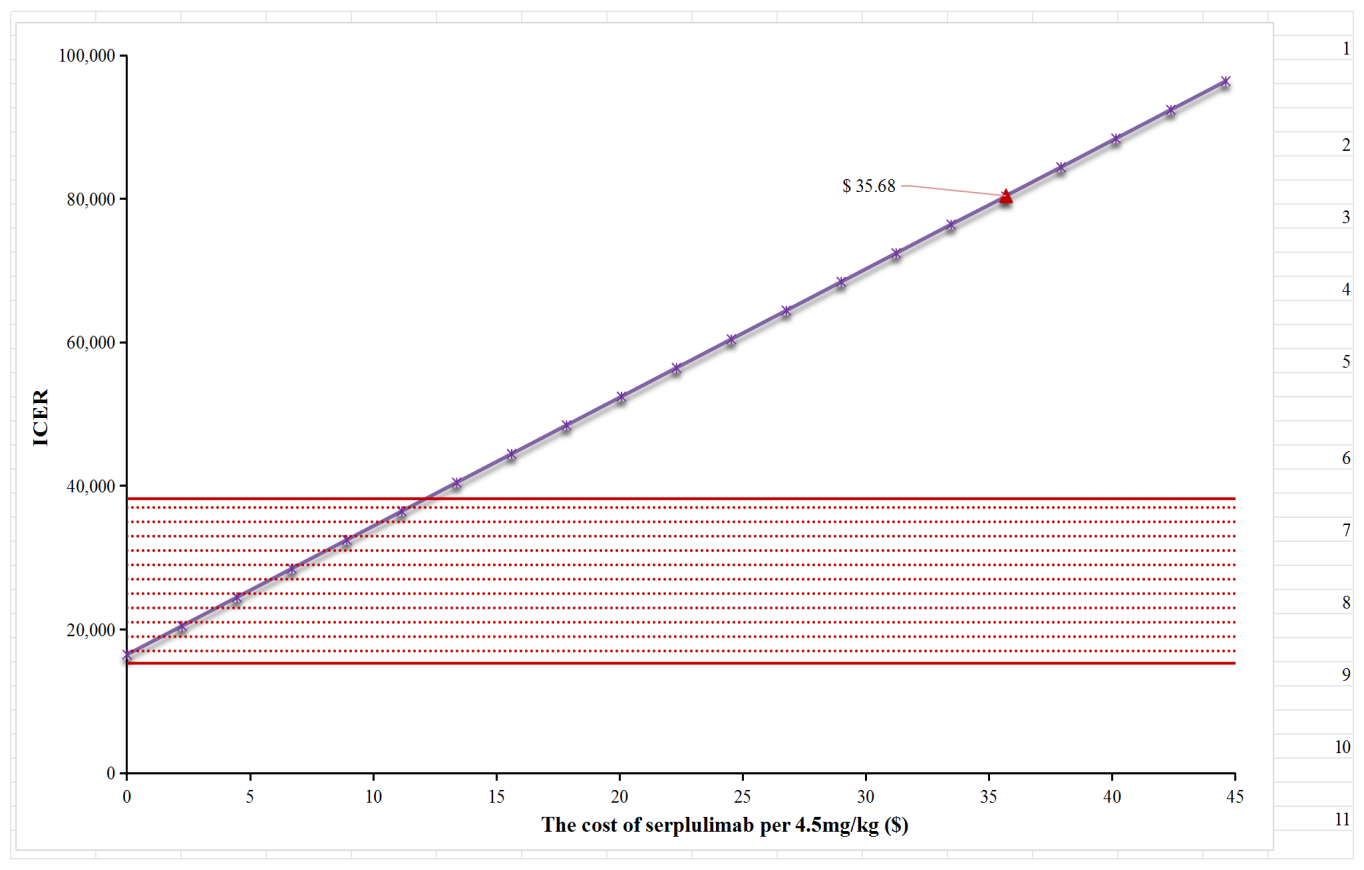


ICER, incremental cost-effectiveness ratio.

**Figure S6. Probabilistic sensitivity analyses results for male patients with ES-SCLC.**


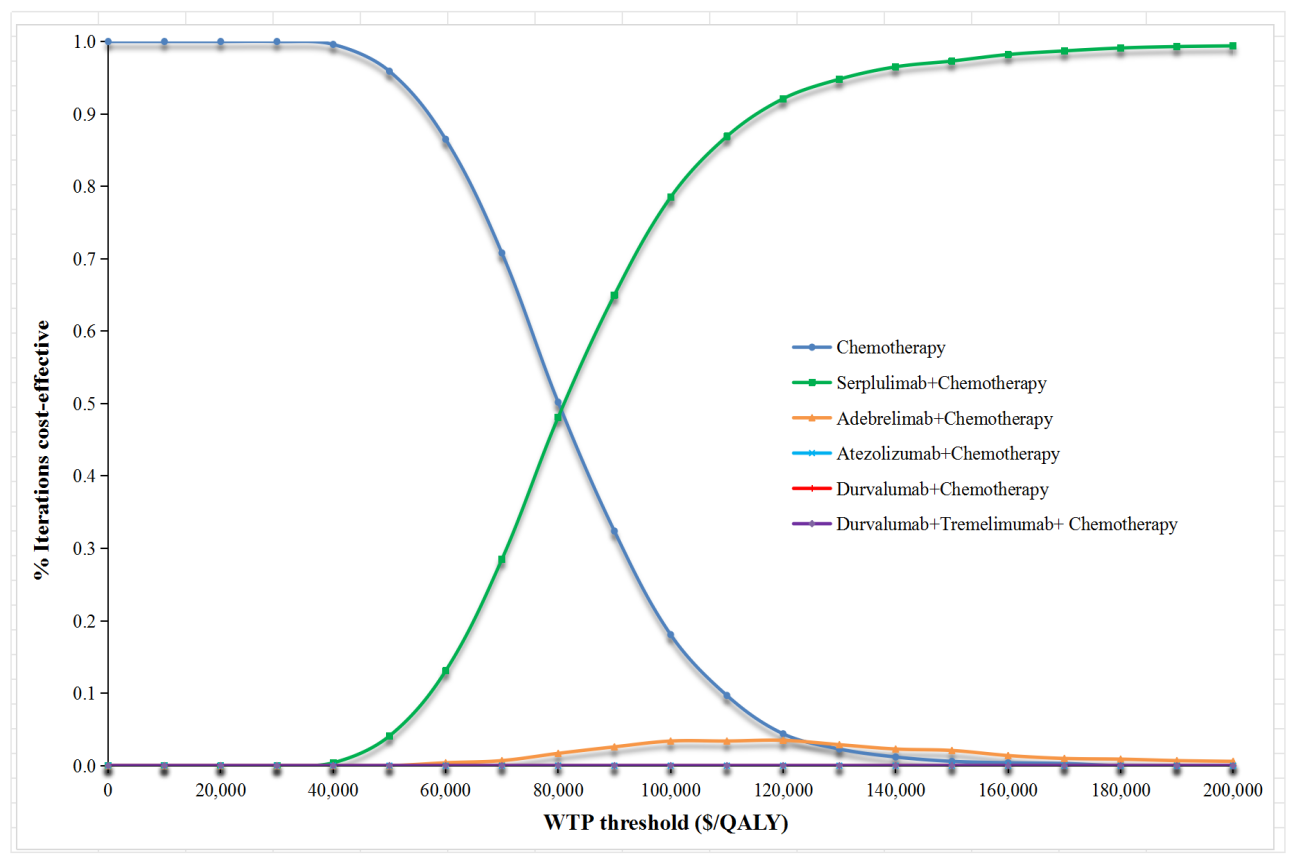


ES-SCLC, extensive-stage small cell lung cancer; WTP, willingness-to-pay; QALY, quality-adjusted life-year.

**Figure S7. Probabilistic sensitivity analyses results for female patients with ES-SCLC.**
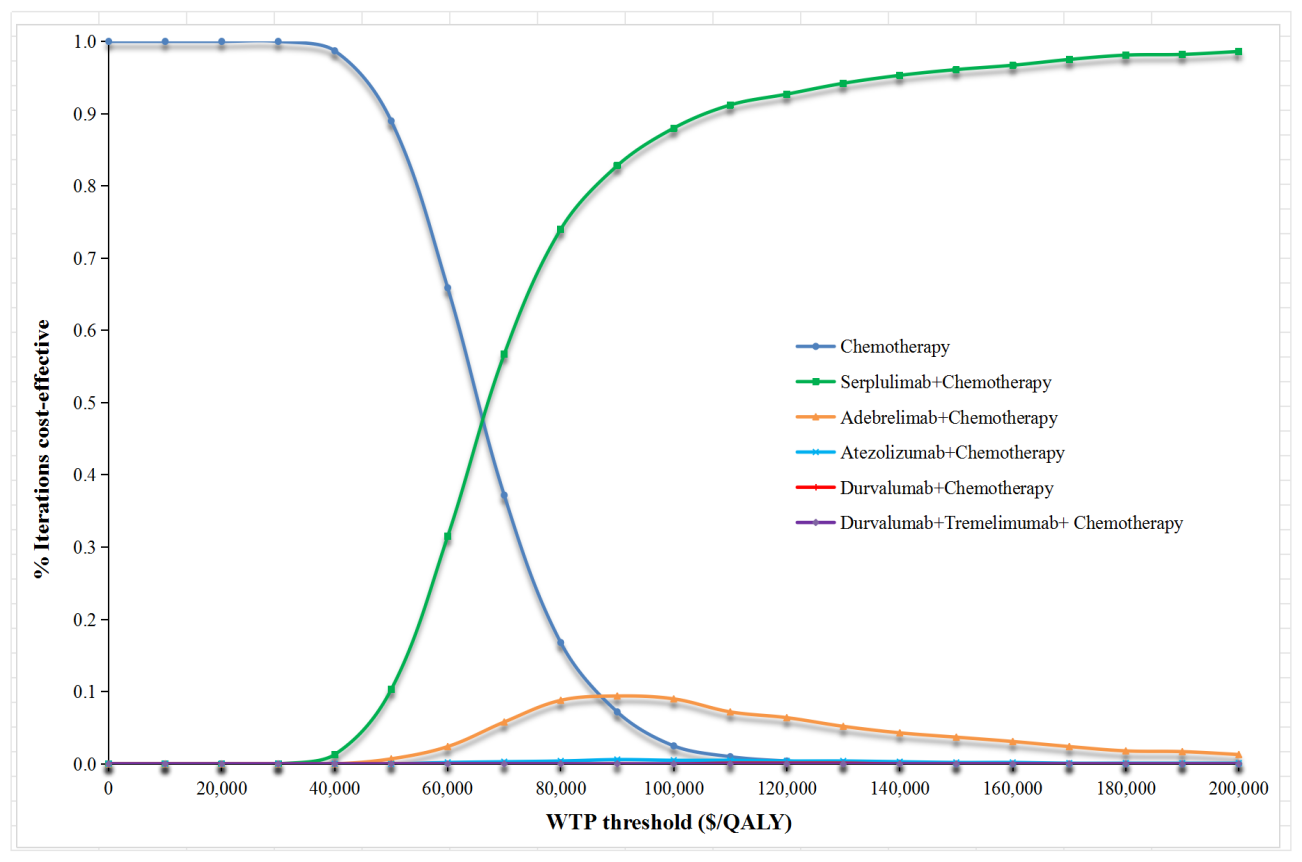


ES-SCLC, extensive-stage small cell lung cancer; WTP, willingness-to-pay; QALY, quality-adjusted life-year.

**Figure S8. Probabilistic sensitivity analyses results for age≥65 patients with ES-SCLC.**
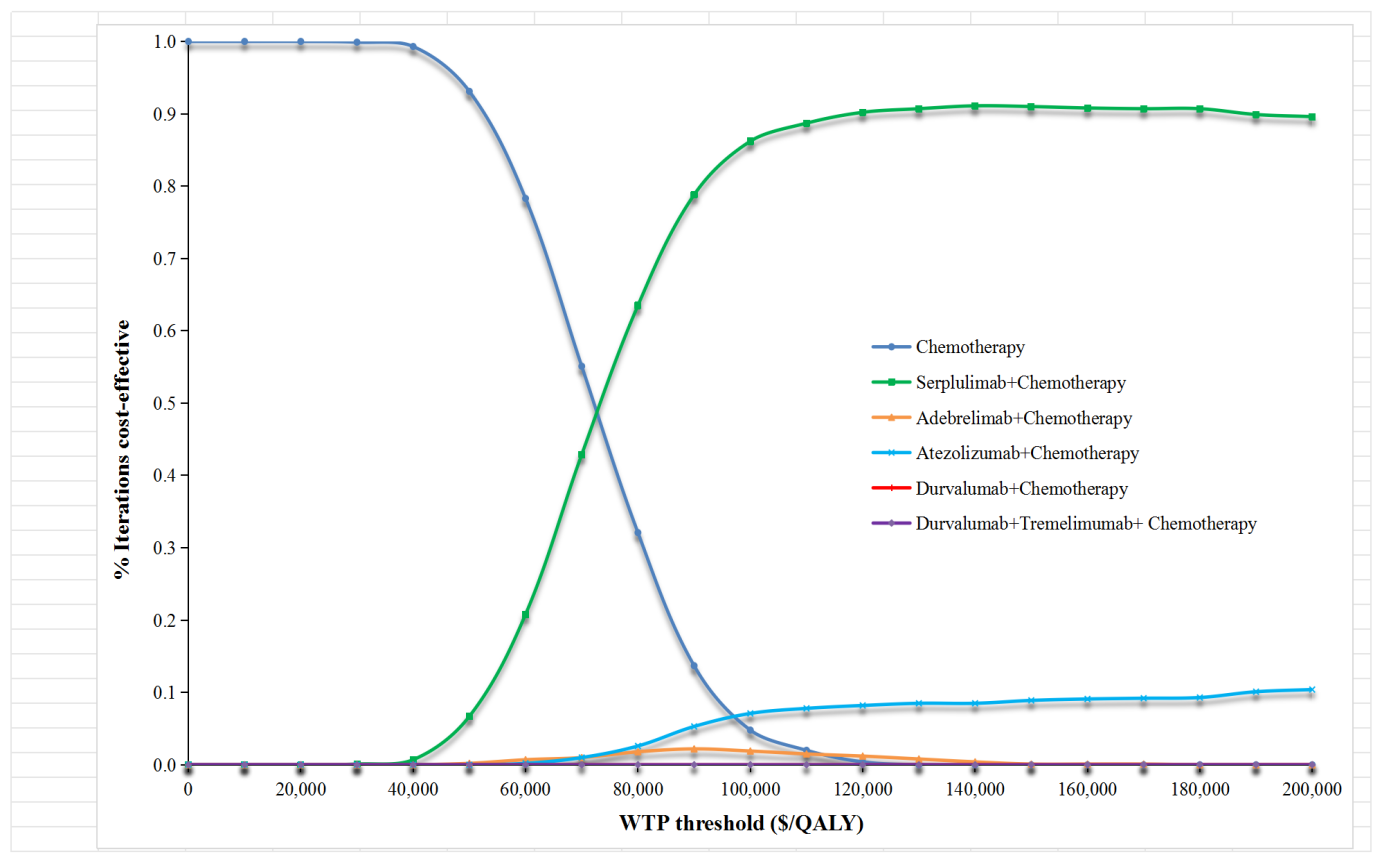


ES-SCLC, extensive-stage small cell lung cancer; WTP, willingness-to-pay; QALY, quality-adjusted life-year.

**Figure S9. Probabilistic sensitivity analyses results for age<65 patients with ES-SCLC.**
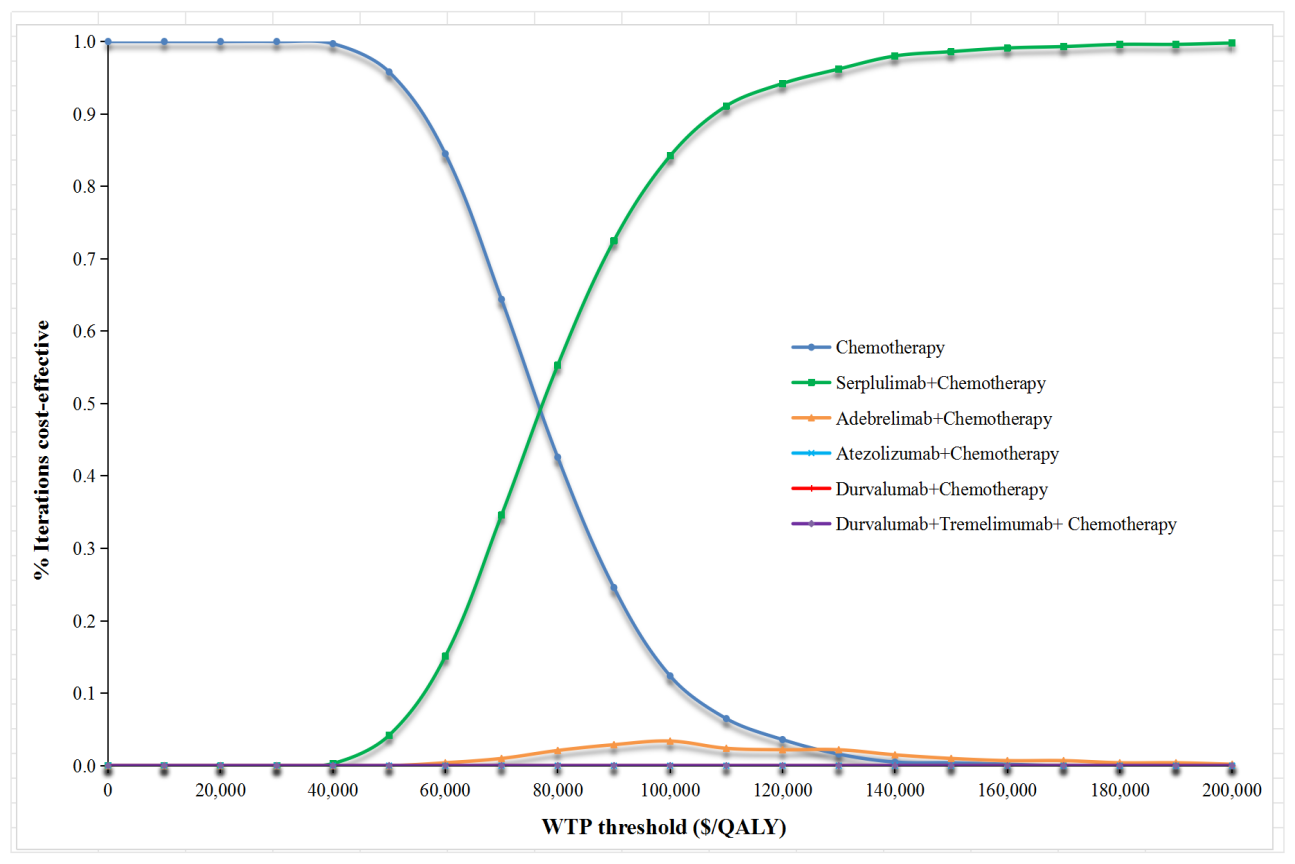


ES-SCLC, extensive-stage small cell lung cancer; WTP, willingness-to-pay; QALY, quality-adjusted life-year.

**Figure S10. Probabilistic sensitivity analyses results for ES-SCLC patients with ECOG performance status score of 0.**
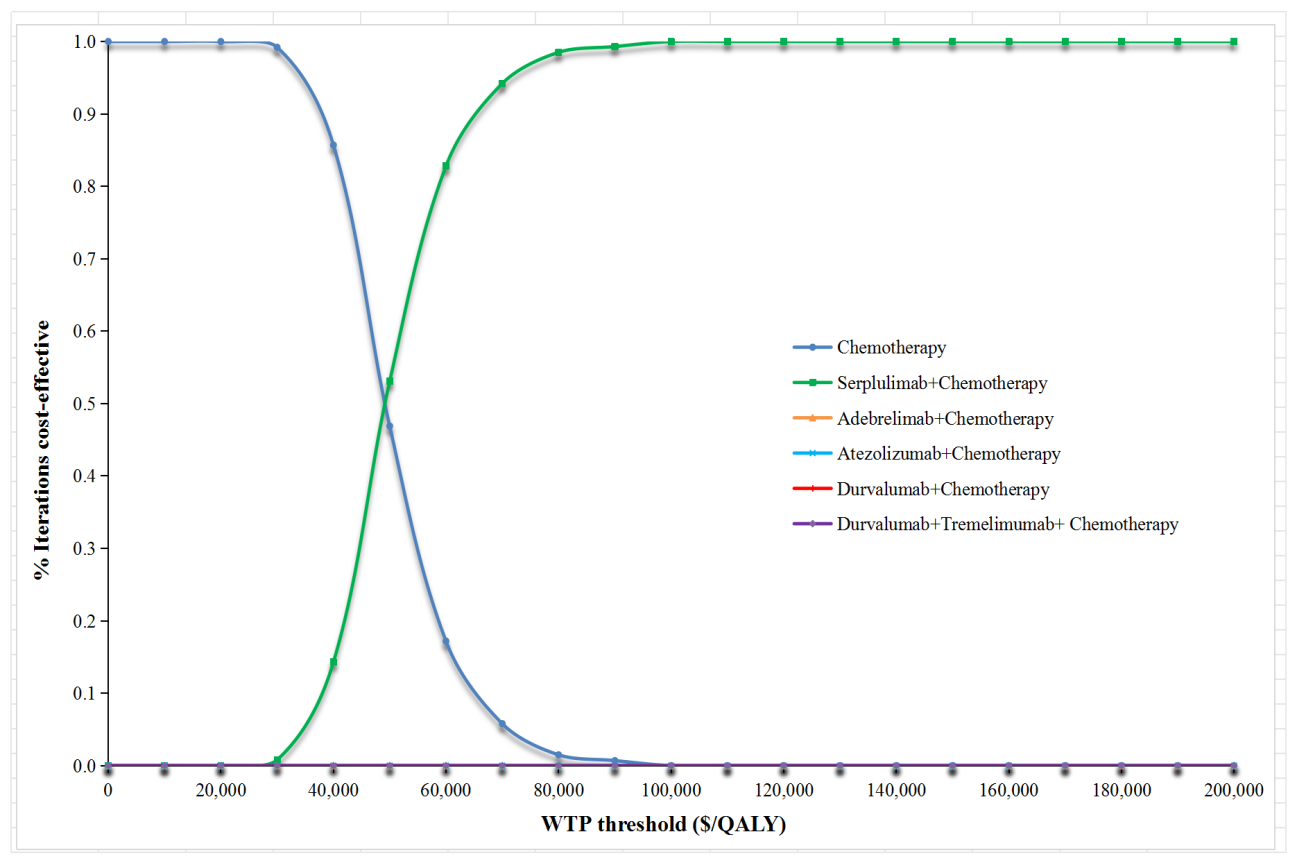


ES-SCLC, extensive-stage small cell lung cancer; ECOG, Eastern Cooperative Oncology Group; WTP, willingness-to-pay; QALY, quality-adjusted life-year.

**Figure S11. Probabilistic sensitivity analyses results for ES-SCLC patients with ECOG performance status score of 1.**


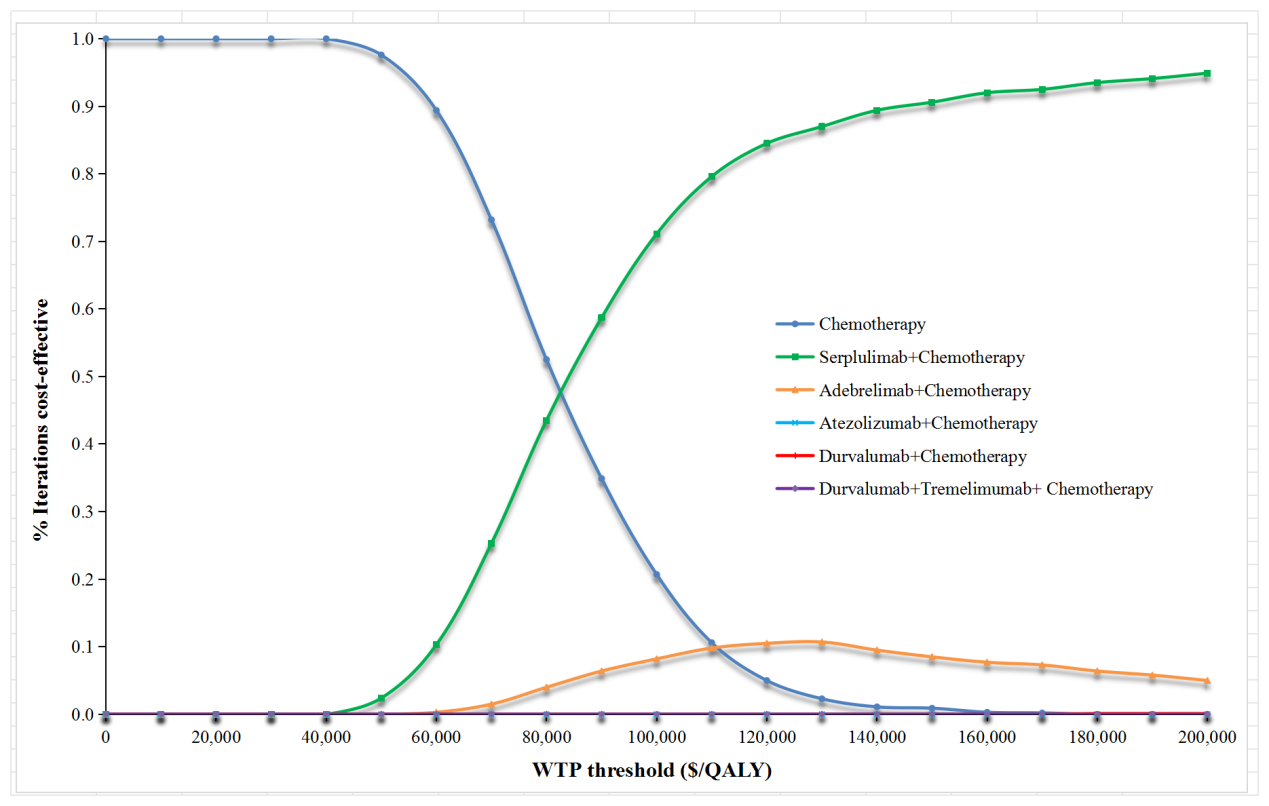
 ES-SCLC, extensive-stage small cell lung cancer; ECOG, Eastern Cooperative Oncology Group; WTP, willingness-to-pay; QALY, quality-adjusted life-year.

**Reference**

1. Wang J, Zhou C, Yao W, et al. Adebrelimab or placebo plus carboplatin and etoposide as first-line treatment for extensive-stage small-cell lung cancer (CAPSTONE-1): a multicentre, randomised, double-blind, placebo-controlled, phase 3 trial. Lancet Oncol. 2022;23(6):739-747.
2. Horn L, Mansfield AS, Szczęsna A, et al. First-Line Atezolizumab plus Chemotherapy in Extensive-Stage Small-Cell Lung Cancer. N Engl J Med. 2018;379(23):2220-2229.
3. Paz-Ares L, Dvorkin M, Chen Y, et al. Durvalumab plus platinum-etoposide versus platinum-etoposide in first-line treatment of extensive-stage small-cell lung cancer (CASPIAN): a randomised, controlled, open-label, phase 3 trial. Lancet. 2019;394(10212):1929-1939.
4. Goldman JW, Dvorkin M, Chen Y, et al. Durvalumab, with or without tremelimumab, plus platinum-etoposide versus platinum-etoposide alone in first-line treatment of extensive-stage small-cell lung cancer (CASPIAN): updated results from a randomised, controlled, open-label, phase 3 trial. Lancet Oncol. 2021;22(1):51-65.
5. Cheng Y, Han L, Wu L, et al. Effect of First-Line Serplulimab vs Placebo Added to Chemotherapy on Survival in Patients With Extensive-Stage Small Cell Lung Cancer: The ASTRUM-005 Randomized Clinical Trial. JAMA. 2022;328(12):1223-1232.
6. Kang S, Wang X, Zhang Y, Zhang B, Shang F, Guo W. First-Line Treatments for Extensive-Stage Small-Cell Lung Cancer With Immune Checkpoint Inhibitors Plus Chemotherapy: A Network Meta-Analysis and Cost-Effectiveness Analysis. Front Oncol. 2022;11:740091. doi: 10.3389/fonc.2021.740091.
7. The society of chemotherapy, Chinese Anti-Cancer Association; Committee of Neoplastic Supportive-care (CONS), China Anti-Cancer Association. Consensus on the clinical diagnosis, treatment, and prevention of chemotherapy- induced neutropenia in China (2019 edition). Chin J Clin Oncol, 2019,46(17):876-882.
8. Committee of Neoplastic Supportive-care (CONS), China Anti-Cancer Association. Chinese Expert Consensus on Management of Thrombocytopenia in Cancer Patients with Liver Injury (2022 Edition). Cancer Res Prev Treat, 2023,50(3): 211-223.
9. The society of chemotherapy, Chinese Anti-Cancer Association; Committee of Neoplastic Supportive-care (CONS), China Anti-Cancer Association. A consensus on the clinical diagnosis, treatment, and prevention of cancer- and chemotherapy-related anemia in China (2019 edition) Chin J Clin Oncol, 2019, 46(17):869-875.
10. The society of chemotherapy, Chinese Anti-Cancer Association; Committee of Neoplastic Supportive-care (CONS), China Anti-Cancer Association. Chinese experts’ consensus on the prevention and the treatment of nausea and vomiting related to anti-cancer drug treatment (2022version). Natl Med J China, 2022,102(39):3080-3094.
11. Chinese Society of Nutritional Oncology, Medical Nutrition Industry Branch of National Association of Health Industry and Enterprise Management, Tumor Nutrition and Therapy Branch of Zhejiang Medical Association. Expert consensus on the nutritional diagnosis and treatment of appetite loss in cancer patients. Electron J Metab Nutr Cancer, 2022, 9(3):312-319.
12. Writing committee of expert consensus on the medication therapy management pathway in hypertensive patients.Expert consensus on the medication therapy management pathway in hypertensive patients. Clinical Medication Journal,2022,20(1):1-24.
13. Lung Cancer Group of Chinese Thoracic Society. Expert consensus on the diagnosis and treatment of immune checkpoint inhibitor-associated pneumonia.Chin J Tubere Respir Dis，2019,42(11):820-825.
14. Expert Committee of Integrated Chinese and Western Medicine,Chinese Society of Clinical Oncology (CSCO).Expert consensus on diagnosis and treatment of Bone marrow suppression caused by anti-tumor drugs by integrated traditional and western medicine.Chinese Clinical Oncology,2021,26(11):1020-1027.
15. Institute for clinical and economic review. Treatment options for advanced non-small cell lung cancer:effectiveness, value and value-based price benchmarks. Final evidence report and meeting summary, 2016,11:1.
16. Delanoy N, Michot JM, Comont T, et al. Haematological immune-related adverse events induced by anti-PD-1 or anti-PD-L1 immunotherapy: a descriptive observational study. Lancet Haematol. 2019 Jan;6(1):e48-e57.
17. Borghaei H, Paz-Ares L, Horn L, et al. Nivolumab versus Docetaxel in Advanced Nonsquamous Non-Small-Cell Lung Cancer. N Engl J Med. 2015 Oct 22;373(17):1627-39.
18. Msaouel P, Oromendia C, Siefker-Radtke AO, et al. Evaluation of Technology-Enabled Monitoring of Patient-Reported Outcomes to Detect and Treat Toxic Effects Linked to Immune Checkpoint Inhibitors. JAMA Netw Open. 2021 Aug 2;4(8):e2122998.
19. Brahmer J, Reckamp KL, Baas P, et al. Nivolumab versus Docetaxel in Advanced Squamous-Cell Non-Small-Cell Lung Cancer. N Engl J Med. 2015,373(2):123-35.
20. Hong Kong Jimin Pharmaceutical. Imjudo (tremelimumab). 2023 <https://www.pidrug.com/>. Accessed March 18 2023.
21. Health industry Big data platform. Bid winning price of drugs. <https://db.yaozh.com/yaopinzhongbiao> Accessed March 12, 2023.
22. Luo X, Liu Q, Zhou Z, et al. Cost-Effectiveness of Bevacizumab Biosimilar LY01008 Combined With Chemotherapy as First-Line Treatment for Chinese Patients With Advanced or Recurrent Nonsquamous Non-Small Cell Lung Cancer. Front Pharmacol. 2022;13:832215.
23. Shen Y, Wu B, Wang X, Zhu J. Health state utilities in patients with advanced non-small-cell lung cancer in China. J Comp Eff Res. 2018;7(5):443-452. doi: 10.2217/cer-2017-0069.
24. Research Group of China Pharmacoeconomics Evaluation Guideline. China Guidelines for Pharmacoeconomic Evaluations (2020 Edition). Chinese Pharmaceutical Association; 2020, page:1-25.
25. Central People's Government of the People's Republic of China. The State Council Office of the People's Republic of China held a press conference on the “Report on the Nutrition and Chronic Disease Status of Chinese Residents (2020)”. <https://www.gov.cn/xinwen/2020-12/23/content_5572785.htm> Accessed March 12, 2023.
